# Transcriptional analysis of degenerate strain *Clostridium beijerinckii* DG-8052 reveals a pleiotropic response to CaCO_3_-associated recovery of solvent production

**DOI:** 10.1038/srep38818

**Published:** 2016-12-14

**Authors:** Shengyin Jiao, Yan Zhang, Caixia Wan, Jia Lv, Renjia Du, Ruijuan Zhang, Bei Han

**Affiliations:** 1 School of Public Health, Health Science Center, Xi’an Jiaotong University, Xi’an, China; 2 Institute for Genome Sciences, University of Maryland, School of Medicine, Baltimore, Maryland, USA; 3 Department of Bioengineering, University of Missouri Columbia, Missouri, USA

## Abstract

Degenerate *Clostridium beijerinckii* strain (DG-8052) can be partially recovered by supplementing CaCO_3_ to fermentation media. Genome resequencing of DG-8052 showed no general regulator mutated. This study focused on transcriptional analysis of DG-8052 and its response to CaCO_3_ treatment via microarray. The expressions of 5168 genes capturing 98.6% of *C. beijerinckii* NCIMB 8052 genome were examed. The results revealed that with addition of CaCO_3_ 565 and 916 genes were significantly up-regulated, and 704 and 1044 genes significantly down-regulated at acidogenic and solventogenic phase of DG-8052, respectively. These genes are primarily responsible for glycolysis to solvent/acid production (*poR, pfo*), solventogensis (*buk, ctf, aldh, adh, bcd*) and sporulation (*spo0A, sigE, sigma-70, bofA*), cell motility and division (*ftsA, ftsK, ftsY, ftsH, ftsE, mreB, mreC, mreD, rodA*), and molecular chaperones (*grpE, dnaK, dnaJ, hsp20, hsp90*), etc. The functions of some altered genes in DG-8052, totalling 5.7% at acidogenisis and 8.0% at sovlentogenisis, remain unknown. The response of the degenerate strain to CaCO_3_ was suggested significantly pleiotropic. This study reveals the multitude of regulatory function that CaCO_3_ has in clostridia and provides detailed insights into degeneration mechanisms at gene regulation level. It also enables us to develop effective strategies to prevent strain degeneration in future.

Solventogenic *Clostridium* species are unique natural occurring microorganisms capable of performing acetone-butanol-ethanol (ABE) fermentation with butanol as the major metabolite. One challenge with industrial ABE fermentation by solventogenic *Clostridia* is strain degeneration due to repeated subculture or continuous fermeantion, which often leads to significantly reduced fermentation efficiency and even predetermined fermentation^1^,^2^. Another cause of strain degeneration is bacteriophage infection due to which many industrial ABE fermentation processes were compromised in early 20th century^3^. Over the past three decades, extensive research were carried out to find solutions for overcoming *Clostridia* strain degeneration. It was reported that addition of sodium acetate to MP2 medium could prevent degeneration in *Clostridium beijerinckii* NCIMB 8052 and BA101, a solvent-hyperproducing mutant derived from 8052^4^; Recently, in hyper-butanol producing *C. acetobutylicum* JB200, the integrated gas stripping to remove product during fermentation was found to increase butanol productivity without culture degeneration^5^,^6^.

Degenerate solventogenic *Clostridium* strains are known to partially or completely lose their capability to catabolize acetic acid and butyric acid generated at acidogenic phase for ABE production at the following solventogenic phase. The resulting acid accumulation exerts a strong stress to degenerate strains, eventually leading to cell death/lysis and cease of solvent production^7^,^8^. The degeneration mechanism varies among *Clostridium* strains. *C. acetobutylicum* (ATCC 824) is degenerated as a result of the loss of the mega-plasmid pSOL1 which harbours *sol* operon (*aad*-*ctfA*-*ctfB*) expressing alcohol/aldehyde dehydrogenase and CoA transferase responsible for the acid uptake^9^. For *C. saccharoperbutylacetonicum*, a degenerate strain is identified with a defect in genes encoding for enzymes responsible for the NADH formation from the catabolism of pyruvate^10^. *C. beijerinckii* NCIMB 8052 has all the genes responsible for ABE fermentation in the genome, and more degenerate variants of *C. acetobutylicum* was found than *C. acetobutylicum* (ATCC 824)^4^. During ABE fermentation, CaCO_3_ has been shown to stimulate sugar utilization, butanol production, and butanol tolerance^11^. We reported previously that CaCO_3_ could enhance ABE fermentation in both the wild type *C. beijerinckii* strain (NCIMB 8052) and degenerate one. The proteomic analysis results showed that key cellular processes, such as sugar transport, butanol tolerance, and solventogenesis were influenced by the addition of CaCO_3_^12^,^13^.

The objective of this study is to elucidate the regulation mechanism of calcium on the degenerate strain of *C. beijerinckii* NCIMB8052 by transcriptional analysis. We first compared the differences between the whole genomes of DG-8052 and WT-8052 using a genome resequencing strategy, and then compared the abundance of transcription of genes in CaCO_3_ treated and untreated DG-8052, especially in ABE fermentation, cell division and spore forming. This study is expected to provide possible strategies in engineering *C. beijerinckii* NCIMB 8052 to prevent strain degeneration and improve ABE fermentation.

## Results and Discussion

### Effect of CaCO_3_ on cell growth of DG-8052

**Figure 1:**
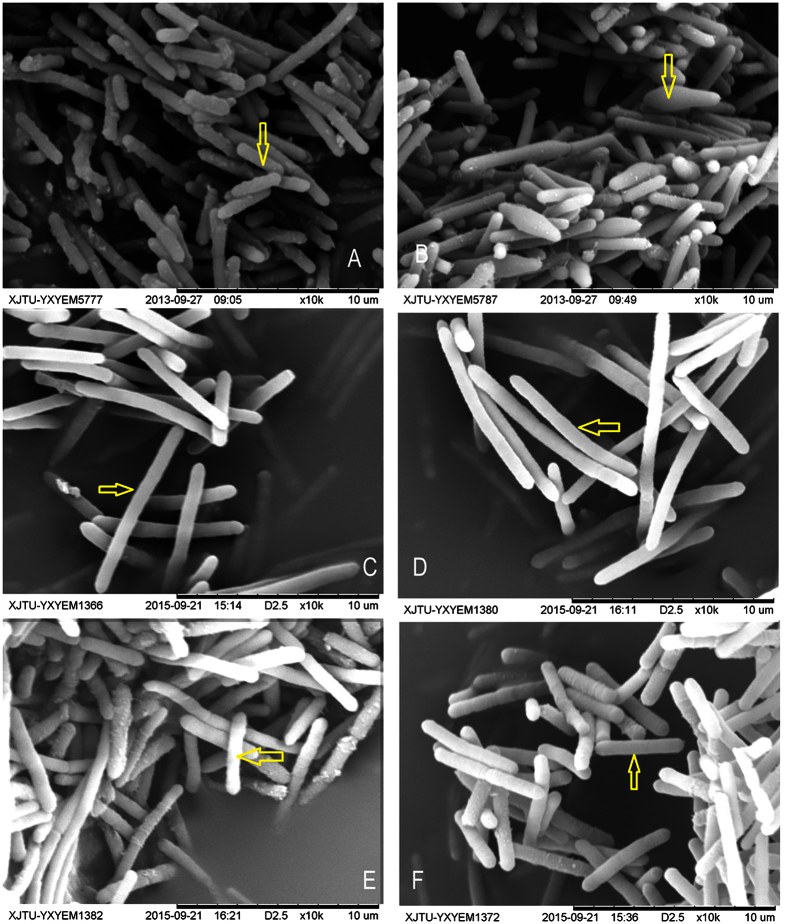
Electron micrograph of *C. beijerinckii* cells grown on P2 medium with and without CaCO_3_. WT-8052 strain cultured at 12 h (**A**) and 24 h (**B**); DG-8052 strain at 12 h (**C**) and 24 h (**D**); DG-8052 cells strain cultured with 4 g/L CaCO_3_ at 12 h (**E**) and 24 h (**F**). The typical cells were indicated by yellow arrows.

In solventogenic *Clostridium* species, the spore development (sporulation) is usually accompanied with solvent production. Usually, during the acidogenic phase, cells in most cultures consist of straight, short or long rods with round ends (Fig. 1A). Towards the end of exponential growth the rod-shaped cells typically begin to accumulate granulose, assuming a swollen cigar-shaped clostridia form, and produce extracellular slime or capsules (Fig. 1B)^14^,^15^. In contrast, DG-8052 showed a large proportion of non-split and elongated straight rod cells (8–10 μm × 0.8 μm at acidogenic phase, 8–12 μm × 0.8 μm at solvetogenic phase) (Fig. 1C and D). DG-8052 only retained little capability of solvent production, having very small amounts of solvents produced (0.10 g/l acetone, 0.19 g/l ethanol and 0.58 g/l butanol) during 48 hours or longer culture period^13^. Similar phenomena were observed with a degenerate strain *C. acetobutylicum* M5 which lost the capability of sporulation and solvent production^8^. Our previous study indicated that CaCO_3_ played an important role in improving the performance of DG-8052 on cell growth, glucose utilization, and solventogenesis. With the addition of 4 g/l CaCO_3_, the solventogenesis was partially recovered, reaching 1.94 g/l acetone, 0.25 g/l ethanol and 5.44 g/l butanol throughout the fermentation period of 60 h^13^. In addition, the DG-8052 growing in the media supplement with CaCO_3_ appeared to have short rod-like morphology (2–6 μm × 0.8 μm) (Fig. 1E and F), resembling those of the wide type one much, especially at the exponential phase. The morphological change along with partially restored solventogenic capability indicated that CaCO_3_ was beneficial to *C. beijerinckii* DG-8052.

### Overall gene transcription dynamics

**Figure 2:**
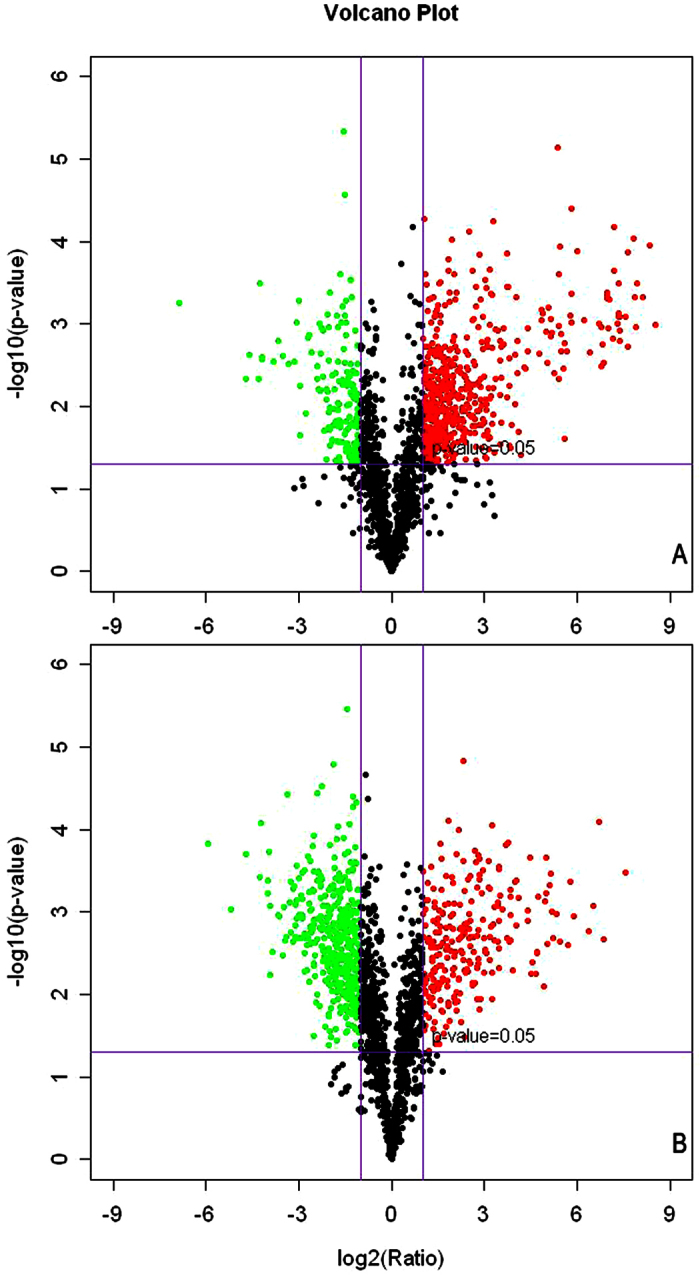
Comparison of gene expression in DG-8052 in P2 medium with CaCO_3_ vs DG-8052 in P2 medium. Cells from both strains were taken at 12 h and 24 h to compare transcriptome profiles at acidogenic (**A**) and solventogenic (**B**) phases, respectively. Genes with significantly differential expression (fold change >2 and p-value < 0.05) between the two strains were shown in green (down-regulated) and red (up-regulated) while the others with non-significantly differential expression were shown in black.

**Figure 3:**
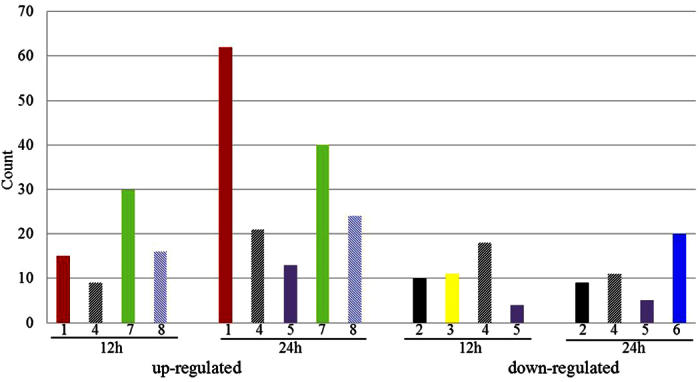
Counts of differentially expressed genes by KEGG pathway categories. 1, carbohydrate metabolism; 2, energy metabolism; 3, nucleotide metabolism; 4, amino acid metabolism; 5, metabolism of other amino acid; 6, metabolism of co-factors and vitamins; 7, membrane transporter; 8, signal transduction.

Transcriptome profiles of DG-8052 treated with and without CaCO_3_ were compared by microarray analysis. Since ABE fermentation is a biphasic process, gene expressions were compared at acidogenic phase and solventogenic phase, respectively. Total 5168 genes capturing 98.6% of the *C. beijerinckii* NCIMB 8052 genome were examed (Fig. 2). With the addition of CaCO_3_, DG-8052 had 565 and 916 genes significantly up-regulated at acidogenic phase and solventogenic phase, respectively (Additional file Table S3A, S3C). According to the enrichment analysis of GO terms and KEGG pathway, these genes were significantly overexpressed in terms of cellular functions such as amino acid transport and metabolism, organic acid biosynthetic process, and bacteria chemotaxis (Table 1, Fig. 3). Significantly down-regulated 704 and 1044 genes at acidogenic phase and solventogenic phase, respectively (Additional file Table S3B, S3D) had primary functions in ion transmembrane transport, ATP synthesis, and oxidative phosphorylation (Table 1, Fig. 3). The COG (Clusters of Orthologous Groups) distribution for these up- and down-regulated genes in the transcriptome was determined (Additional file Table S2). Not all of the regulated genes had known function, and COG analysis revealed that 292 and 425 regulated genes at acidogenisis and solventogensis, respectively, had unspecified functions. The differentially expressed genes are described in detail in the latter sections.

At proteomic level, it was found that 3% detected proteins changed in DG-8052, resulting in corresponding morphological and physiological changes; moreover, the addition of CaCO_3_ partially recovered the metabolism, including solvent production, spore formation, and carbon utilization, and there had only about 0.7% detected proteins changed^12^. Transcriptomic data will help to reveal the responses of this bacterium to CaCO_3_, and expatiate on the mechanisms that CaCO_3_ contributed to the up-regulation of ABE fermentation in degenerate *Clostridium* strain.

### Solvent production

CaCO_3_ can improve solvent production in both WT-8052 and DG-8052 strains with higher transcription and expression of solvent producing pathway-related enzymes^12^,^13^. At the acidogenic phase, the addition of CaCO_3_ increased the expression level of genes encoding acids producing enzymes (butyrate kinase Cbei_4609) expressed (+6.05) in the DG-8052 culture (Fig. 3A, Additional file Table S5A). Enough amounts of acetic acid and butyric acid accumulated, then they were re-assimilated and converted into acetyl-CoA and bytyryl-CoA, respectively, by CoA-transferase^16^. The corresponding ORFs of CoA-transferase (Cbei_2040, Cbei_3278, Cbei_3833 and Cbei_3834) showed different transcription. When CaCO_3_ added, Cbei_3833 and Cbei_3834 showed no obviously expressing change at both acidogenisis and solventogensis, while Cbei_2040 and Cbei_3278 showed up-regulated fold change from +3.40, +3.99 (acidogenisis) to +4.69, +12.99 (solventogensis), separately. The regulation mechanism of sharp increased expression of CoA-transferase in DG-8052 with CaCO_3_ is still unknown. It may be because of the effects of Ca^2+^ and the buffering capacity of CaCO_3_ to the fermentation media^12^,^13^. The acetyl-CoA acetyltransferase (Cbei_3630) showed increased transcription while Cbei_0411 showed decreased transcription, and there had 86% similarity in their amino acid sequences, which may cause the different sensitivity to CaCO_3._ The transcription of 3-hydroxybutyryl-CoA dehydrogenase (Cbei_0325), 3-hydroxybutyryl-CoA (Cbei_0324) and butyryl-CoA dehydrogenase (Cbei_0322) was not changed significantly by the addition of CaCO_3_ to the DG-8052 culture.

**Figure 4:**
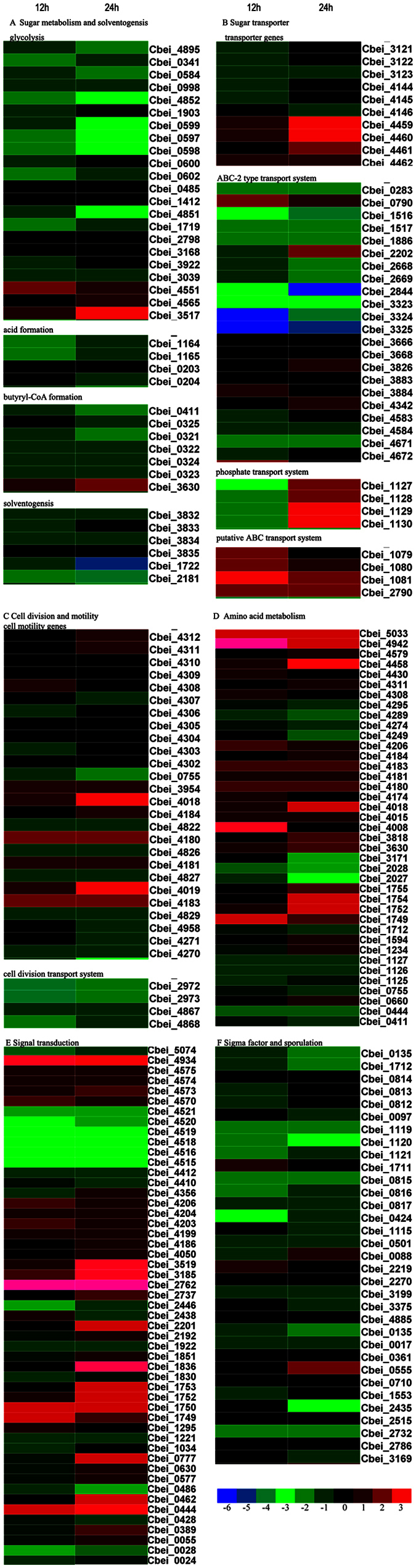
Comparative expression patterns for genes related to (**A**) glycolysis, acidogensis and solventogensis, (**B**) sigma factor and sporulation, (**C**) Cell division and cytoskeleton, (**D**) Sugar transporter/metabolism, (**E**) amino acid biosynthesis/metabolism, (**F**) Signal transduction. Left column is the acidogenisis, and right column is the solventogenisis.

**Figure 5:**
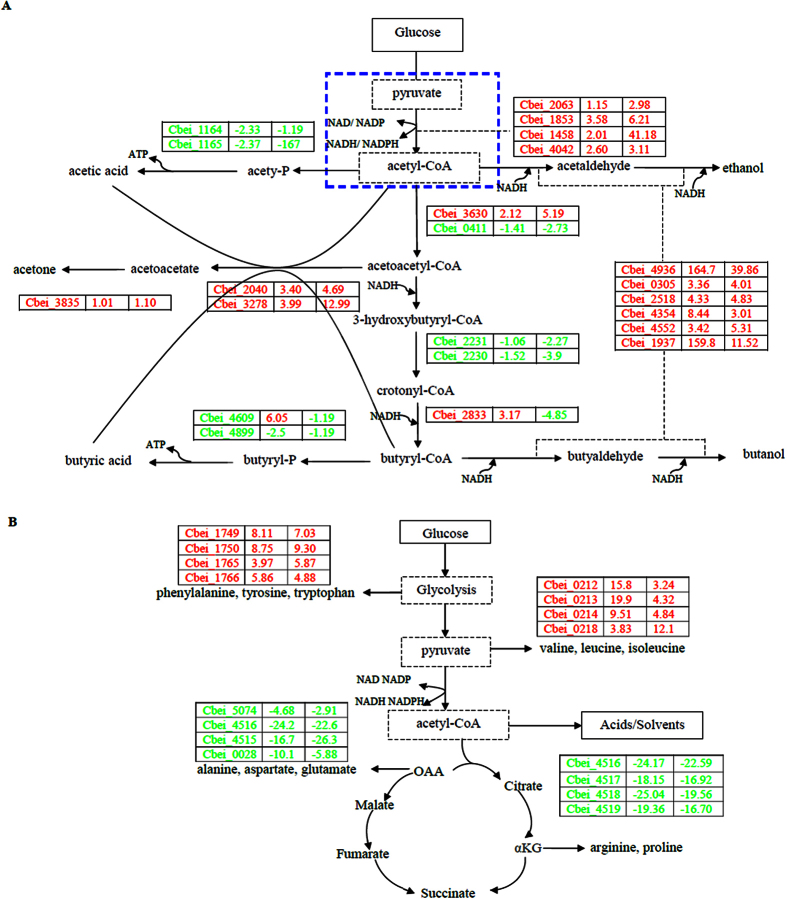
Schematic diagram showing the primary steps in conversion of glucose into fermentation products in *C. beijerinckii* DG-8052. Acids/solvents producing pathway (**A**), amino acids producing pathway (**B**). The fold change (FC) value is indicated directly beside the gene ID of 12 h and 24 h respectively. Text in red and green represents the up-regulated and down-regulated genes, respectively. The blue dash box indicated the synthesis of acetyl-CoA from pyruvate. Detailed enzymes and their associated gene ID can be found in Supplementary Data Table S4A,4C.

It is reported that phosphoenolpyruvate (PEP), pyruvate, and acetyl-CoA form three main key nodes in the flux distribution^17^. In solvetogenic *clostridia*, glucose is converted into pyruvate via glycolysis accompanied with 2 ATP and 2 NADH, and the produced pyruvate is further converted into acetyl-CoA for the production of acetic and butyric acid at acidogenic phase. It had no significant difference in transcription of enzymes responsible for acid production and subsequent conversion to acetyl-CoA and butyryl-CoA between the DG-8052 cells with and without CaCO_3_. Thus, the increased production of solvent may be attributed to either the up-regulated aldehyde dehydrogenase and alcohol dehydrogenase, or the high amount of reaction substrate (acetyl-CoA). We found in DG-8052 supplied with CaCO_3_, pyruvate synthase (Cbei_2063), puryvate/ferredoxin oxidoreductase (Cbei_1853, Cbei_1458, Cbei_4042) were all up-regulated at acidogenisis and solventogensis, and Cbei_1458 had a maximum fold change of 41.18 at solventogenisis (Figs 4 and 5A, Additional file Table S5A), which may result in high concentration of acetyl-CoA. Energy and reducing power (NADH/NADPH) has been reported to have a significant influence on cell growth, metabolism and solvent production^14^. The problem with formation of NADH from pyruvate^10^ was report to lead to degeneration of *C. saccharoperbutylacetonicum*. CaCO_3_ appeared to increase high level of NADH accompanied with increased producing of Acetyl-CoA from pyruvate, which could contribute to some restored functions of DG-8052. Further study is needed to verify the positive effects of CaCO_3_ on the formation of NADH.

Aldehyde dehydrogenase was reported to play key roles in primary alcohol production of *C. beijerinckii* 8052^18^,^19^. Cbei_1722 and Cbei_2181 in DG-8052 with 94–99% amino acid sequence identical to that of *adhA* and *adhB*, showed no change in their expression in response to CaCO_3_. The aldehyde dehydrogenase (Cbei_2518, Cbei_0305 and Cbei_4936) showed up-regulation folds from +4.33, +3.36, +164.7 (acidogenisis) to +4.83, +4.01, +39.86 (solventogensis), separately. Alcohol dehydrogenase (Cbei_4552, Cbei_4354 and Cbei_1937) were also all up-transcripted from +3.42, +8.44, +159.77 (acidogenisis) to +5.31, +3.0 +11.52 (solventogensis), separately. There were 12 aldehyde dehydrogenase encoded genes and 25 alcohol dehydrogenase encoded genes in *C. beijerinckii* NCIMB 8052, suggesting the great potential for the solvent production in this bacterium. Most of these genes were induced at the early acidogenic phase and highly expressed in the solventogenic stage (Additional file Table S5A). Our previous report revealed the broader effects of calcium at the cellular and protein levels on carbohydrate utilization, acids uptake, butanol production and tolerance in ABE production by *C. beijerinckii* NCIMB 8052^12^. The metal-driven stimulatory effects on ABE production were somehow multifactorial and multifunctional due to the complex metabolic network of *clostridia*^20^. The specific functions of different aldehyde dehydrogenase and alcohol dehydrogenase genes in *C. beijerinckii* NCIMB 8052 and different transcription in DG-8052 required further investigation.

### Sugar transporter and metabolism

Our previous report indicated that the DG-8052 could metabolite more glucose when supplied with CaCO_3_ during the batch fermentation. The phosphoenolpyruvate-dependent phosphotransferase system (PTS) is the predominant sugar uptake pathway in *C. beijerinckii*^21^. In *C. beijerinckii* 8052, EI (enzyme I, Cbei_0196) and HPr (heat stable, histidine-phosphorylatable protein, Cbei_1219), phosphorylate the sugar in the cytoplasm, which were all down-regulated in DG-8052 when supplied with CaCO_3_. Except this, other sugar PTS transporters were all not affected, which may point out that in DG-8052, the genes related to carbohydrate metabolism pathways were not impacted by CaCO_3_, or the addition of CaCO_3_ did not affect PTS sugar transportation system (Fig. 4A,B, Additional file Table S5B). In *Cyanobacterium synechocystis* sp. PCC 6803, the sugar catabolic pathways were positive regulated by SigE^22^. Our results showed than compared with DG-8052 without CaCO_3_, SigE (Cbei_1120) got down-regulated by 2.76 and 4.79 folds in DG-8052 with CaCO_3_ (Additional file Table S5D). And the down-regulated SigE may repress the sugar metabolism related genes.

However, not all carbohydrates are accumulated in PTS system, and there were a non-PTS system probably energized by the transmembrane proton gradient in *C. beijerinckii*^23^. In DG-8052 supplied with CaCO_3_, the increased transport of glucose accompanied with increased production of acetic and butyric acids, while those acids were converted into dissociated form and increased the transmembrane proton gradient. In *C. beijercinkii* CC101, which derived from NCIMB8052, the addition of citrate could inhibit cell growth by reducing the cells internal pH and proton motive force, and changing cell membrane permeability^24^. In our research, the supplied CaCO_3_ could convert the undissociated acids into dissociated form which reduced the acids stress to cell, while, the produced butyrate and acetate may contribute like citrate did in CC101, which may explain the partial recovery of ABE producing ability in DG-8052.

And it showed the non-PTS transport system contributed significantly to glucose uptake at solventogenic phase but not acidogenic phase^23^. In the present study we found in DG-8052, the putative ABC transport system including phosphate binding protein (Cbei_1127), phosphate ABC transporter permease (Cbei_1128, Cbei_1129), phosphate ABC transporter ATPase (Cbei_1130) were down-regulated at acidogenisis, but up-regulated at solventogenisis when supplied with CaCO_3_ (Fig. 4B, Additional file Table S5B). It may contribute to the higher transport of sugar in DG-8052 with presence of CaCO_3_.

Generally, glycolysis genes encoding enzymes for conversion of glucose to pyruvate were expressed at high levels to provide enough ATP for cell growth. At the generic level, expression of glycolysis pathway enzymes in DG-8052 cell were down regulated by adding CaCO_3_, except ROK family protein (Cbei_4565, Cbei_3517) and fructose-bisphosphate aldolase (Cbei_4551) (Fig. 4A). Compared with the proteomic data, the translation of those down-transcript genes had no significant change in DG-8052 with and without CaCO_3_^13^. It may indicate the sugar could be transported into the cell via non-PTS system (ABC transporter), and metabolized by glycolysis.

### Amino acid biosynthesis/metabolism

Amino acids are key metabolites that reflect the intercellular status^25^. In general, biosynthesis of phenylalanine, tyrosine and tryptophan (cbe00400), valine, leucine and isoleucine (cbe00290) was apparently up-regulated (Fig. 3, Table 1, Additional file Table S5C). Genes involved in phenylalanine, tyrosine, tryptophan biosynthesis (Cbei_1749, Cbei_1750, Cbei_1752, Cbei_1753, Cbei_1754, Cbei_1755) were up-regulated about 10-fold at solventogenisis. Genes involved in valine, leucine and isoleucine biosynthesis (Cbei_0212, Cbei_0213, Cbei_0217) were up-regulated about 20-fold at acidogenesis, and 3–18 fold at solventogenisis, along with their transporter genes (ABC transporter genes, Cbei_1762, Cbei_1763, Cbei_1764, Cbei_1766, Cbei_1767, 4–6 fold increased), which are the side pathways from glycolysis and pyruvate, separately.

The biosynthesis of arginine and proline (cbe00330), alanine, aspartate and glutamate (cbe00250) was down-regulated. Genes involved in alanine, aspartate and glutamate (Cbei_4516, Cbei_4515, Cbei_5074) were down-regulated about 16 to 26-fold both at acidogensis and solventogenisis, which is the by-pathway of TCA. In *C. acetobutylicum*, arginine biosynthesis genes were often found to be strongly up-regulated under butyric acid stress and down-regulated under butanol stress, and it was suggested that arginine expression played an important role in metabolite stress^26^,^27^. In both WT-8052 and DG-8052 strains supplied with CaCO_3_, the produced butyric acid was neutralized to form butyrate by CaCO_3_, resulting in a balance in butyric acid and butyrate and decreased acid stress as well as improved butanol production^12^,^13^. It could be explained that biosynthesis of arginine and proline was down-regulated during the whole fermentation in DG-8052 when supplied with CaCO_3._ Additionally, the prevalence of this pathway suggests that wide type *C. beijerinckii* NCIMB 8052 was accumulating proline intracellularly to act as an osmoregulator, as was shown to occur previously in other bacteria, particularly gram-positive strains^27^. However, In DG-8052, the addition of CaCO_3_ broke down this osmoregulating balance, and the genes related in proline metabolism (cbe00330) were down-regulated.

With addition of CaCO_3_, DG-8052 could move towards solventogenisis, and begin to form spores, during which cells required more ATP, NADH and intermediate products. The biosynthesis of amino acid is the most energetically expensive pathway. On the other hand, reduced availability of metabolic energy might be involved in the reduced supply of the ‘expensive’ amino acids^28^. Therefore, DG-8052 cells metabolized glucose into pyruvate, which was in turn converted into acetyl-CoA, followed by entering acids/solvents pathway instead of TCA cycle, which may cause the up-regulation of phenylalanine, tyrosine, tryptophan, valine, leucine and isoleucine, and down-regulation of arginine, proline, alanine, aspartate and glutamate (Fig. 5B).

### Sporulation

The initiation of sporulation in *C. beijerinckii* 8052 was concurrent with the onset of solventogenisis. Sporulation is generally regulated by the transcription factor Spo0A both in bacilli and clostridia, which act as a switch from vegetative growth to sporulation^29^,^30^. Our results revealed the expression of *spo0A* (Cbei_1712) at acidogenic phase was down-regulated by 1.5 folds and further repressed at solventogenic phase 2.31 folds (Fig. 4F). Phosphorylated *spo0A* has been reported to activate transcription of some genes and represses transcription of other genes, which include the soloventogenic operon and multiple sporulation sigma factor genes in *C. acetobutylicum*^31^. Spo0A represses *abrB* gene, a repressor of sporulation genes, and initiates transcription of sporulation genes in *Bacillus sublitis* and *C. acetobutylicum*^32^. In DG-8052, there were 6 ORFs annotated as genes encoding AbrB family transcript regulator (Cbei_0088, Cbei_1757, Cbei_2219, Cbei_2270, Cbei_3375, Cbei_4885). When supplied with CaCO_3_, Cbei_2219 had a 2.24 folds increase at acidogenic phase, while Cbei_0088 had a 3.06 folds increase at solventogenic phase, and Cbei_2270 had a 5.07 folds decrease at solventogenic phase. Although DG-8052 cells supplied with CaCO_3_ advanced its growth to solventogenisis, no complete spores were formed. We speculate that the incomplete sporulation was caused by down-regulation of *spo0A* and different regulation of AbrB family transcript regulators.

Sigma factor SigH, encoded by *spo0H*, is the earliest acting sporulation associated sigma factor in *B. subtilis* that controls genes in the transition phase^33^, and SigH regulates the expression of *spo0A*^34^. In this study, the expression of sigma-70 (Cbei_0135, Cbei_3569, Cbei_3576, Cbei_3675) were significant down-regulated in DG-8052 supplied with CaCO_3_ at acidogenisis and at solventogenisis, where Cbei_3569 and Cbei_3576 was highly repressed by −14.28, −14.65 at 12 h and −7.33, −7.60 at solventogenisis (Additional file Table S5D), which may result from the repressed expression of SigH.

As we reported before, when supplying CaCO_3_ to WT- 8052, the proteomic analysis showed a great increase in the expression of heat shock proteins (GrpE, +28.1; DnaK, +11.7) at early solventogensis stage^12^. A similar increase in the transcription and expression of heat shock proteins *grpE-dnaK-dnaJ* operon (Cbei_0829, +2.67; Cbei_0830, +4.98; Cbei_0831, +3.52) was observed at solventogensis stage of DG-8052 supplied with CaCO_3_DG-8052. However, there was a down-regulation for this operon at acidogenisis stage (Cbei_0829, −2.10; Cbei_0830, −2.48; Cbei_0831, −1.56). Higher transcription and expression of *grpE-dnaK-dnaJ* operon specific at solventogenesis/sporulation may be due to the fact that CaCO_3_ acts as solventogenesis-inducing substances like a quorum sensing signal and thus contributes to the spore formation^2^.

### Cell division and cytoskeleton

The addition of CaCO_3_ could improve the sugar metabolism, growth and solvent producing to DG-8052, but not full recovery was observed with ABE fermentation by DG-8052. Although the morphology of DG-8052 cells supplied with CaCO_3_ changed from non-split and elongated straight rod shape to short rod cells at acidogenic phase (Fig. 1E and F), it was not same as that of wild-type 8052 Figure (Fig. 1A and B). Incomplete recovery of DG-8052 at the presence of CaCO_3_ treatment was verified by the cell division related genes expression (Fig. 4C,E, Additional file Table S5E). There was a great increase in the expression of cell division related protein FtsH (Cbei_3037), with 13.9 folds at 12 h, 16.6 folds at 24 h. In *B. subtilis*, FtsH accumulates in the mid-cell septum during vegetative cell division and at the onset of sporulation at positions near the cell poles that appear to coincide with future division sites^35^. Cell division protein FtsZ is the first protein to move to the division sites, and is essential for recruiting other proteins that produce a new cell wall between the dividing cells, FtsA and FtsK are large integral membrane proteins that coordinate chromosome segregation and cell division^36^. Our results showed at solventogenesis, FtsZ (Cbei_1118) in CaCO_3_ treated DG-8052 cell was not different from untreated cell, while FtsA (Cbei_1117), FtsK (Cbei_1213), FtsY (Cbei_1173) were all down-regulated for 3.12, −3.88 and −3.01 folds, respectively. All these findings may partially explain the morphology change in CaCO_3_ treated DG-8052 cells.

In gram-positive bacteria, the members of the morphogenetic system include MreB (or Mbl), MreC, MreD, PBP2 and RodA. PBP2 particularly determines rod shape, and RodA is required for the maintenance of the rod cell shape and is essential for the elongation of the lateral wall of the cell^37^. Our results showed MreB (Cbei_0491), MreC (Cbei_0492), MreD (Cbei_0494), PBP2 (Cbei_0491), and RodA (Cbei_3365) were all in down-regulation with fold change of −5.28, −2.1, −2.14, −4.42 and −3.66. respectively, at solventogenic (Additional file Table S4E). In DG-8052 supplied with CaCO_3_, cells were still in rod shape at acidogenic phase, but shorten than DG-8052 cells without CaCO_3_; while at 24 h, DG-8052 cells grew into solventogenic stage and began to from spore by the induction of CaCO_3_ and the rod-shape-determining proteins got repressed.

### Cell motility

The cell motility-related genes encoded products responsible for chemotactic responses and flagellar assembly^38^. Motility-related genes in clostridia and bacilli are usually down-regulated in sporulation cells^31^. In this study, the flagellar/chemotaxis multi-gene cluster (Cbei 4312–4302) showed no significant difference between DG-8052 cells treated with and without CaCO_3_, except the increasing of Cbei_4311 (+2.51) and Cbei_4312 (+2.69). Other genes annotated as motility-related, among them, Cbei_0755 (*cheW*) was down-regulated by 3.31-fold, Cbei_4018 (*cheA*) and Cbei_4019 (*cheW*) were up-regulated by 5.80- and 2.86-fold at 24 h (Fig. 4C). The flagellar motility related genes *flgB* (Cbei_4271), *flgC* (Cbei_4270) were down-regulated at 24 h. And it was reported in *B. subtilis* the expression of the flagellar/chemotaxis operon is negatively regulated by Spo0A^39^. Spo0A (Cbei_1712) got down-regulated both at 12 h (−1.5 fold) and 24 h (−2.13 fold), and it seems the regulation effects of Spo0A to the flagellar/chemotaxis genes in *C. beijerinckii* degenerate strain were different from that of *B. subtilis*.

### Validation of gene expression data from microarray analysis by Q-RT-PCR

**Figure 6:**
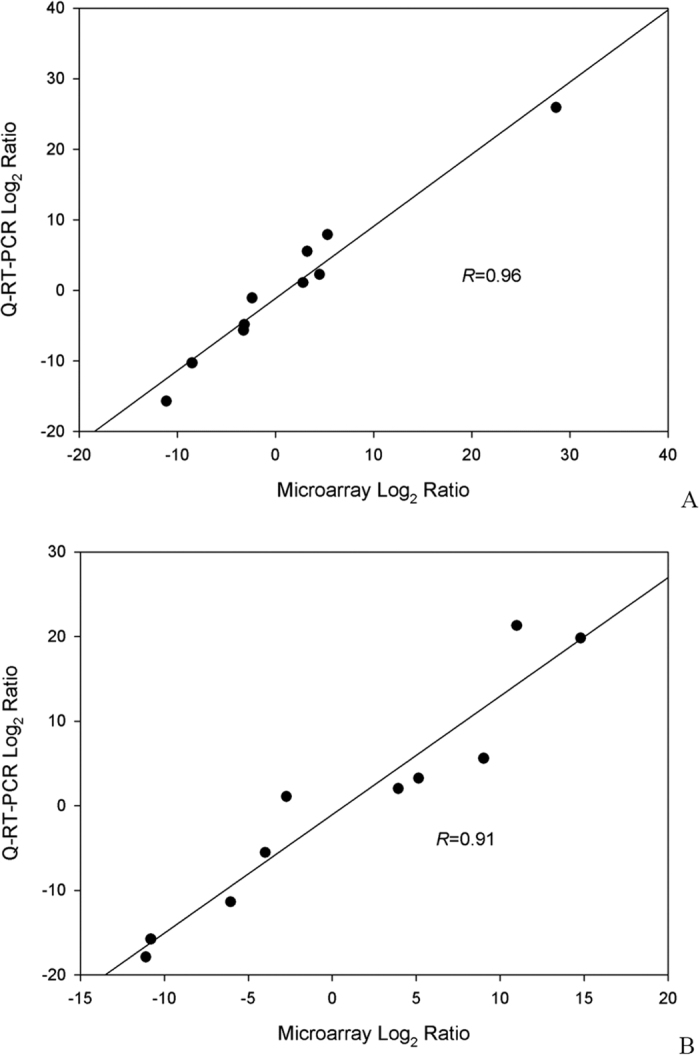
Validation of microarray data from *C. beijerinckii* DG-8052 at two growth stages using Q-RT-PCR. (**A**) acidogenic phase; (**B**) solventogenic phase.

To validate results of gene expressions obtained from microarray analysis, q-RT-PCR was applied to quantify gene expression levels in cultures of DG-8052 supplied with and without CaCO_3_ at the same condition. There were 10 genes at acidogenic phase (12 h) and 10 genes at solventogenic phase (24 h) evaluated. Genes for validation were selected from three blocks: up-regulation (gene expression ratio of DG-8052 with CaCO_3_ to DG-8052 > 2 folds), down-regulation (the gene expression ratio of DG-8052 with CaCO_3_ to DG-8052 < 2 folds) and no remarkable regulation (the gene expression ratio of DG-8052 with CaCO_3_ to DG-8052 between −2 and 2 folds). The results from two expression assays, microarray and Q-RT-PCR, showed that gene expressions in DG-8052 with CaCO_3_ had a high degree of correlation at both acidogenic (R = 0.96) and solventogenic phases (R = 0.91) (Fig. 6, Additional file Figure S1, Table S1). Those genes displayed similar trends and expression levels in the microarray and q-RT-PCR analysis.

### DG-8052 genome resequencing

In order to distinguish the transcriptional modifications associated to the genetic mutations from those associated to the addition of CaCO_3_ during fermentation processes, the DG-8052 was genome resequenced and its results were compared to that of WT-8052. A total of 20 SNPs (17 non-synonymous, 2 premature_stop, 1 intergenic) and 16 InDels (10 coding and 6 non-coding) were found in DG-8052 genome (Table 2). The mutated genes in DG-8052 (microarray data: GSE63671) regulated by CaCO_3_ were Cbei_0769 (extracellular solute-binding protein), Cbei_2569 (DNA mismatch repair protein MutS), Cbei_2653 (3-oxoacid CoA-transferase subunit B), Cbei_1662 (hypothetical protein) and Cbei_2885 (L-lactate transport). It is worth exploring those mutated genes further as they were sensitive to CaCO_3_. While among those genes, there were no general or specific regulators, which may indicate the strain degeneration was not caused by the mutation of them. And the transcriptional changes of DG-8052 were affected by the addition of CaCO_3_ during fermentation.

### Summary

During the growth of *C. beijerinckii* DG-8052, the accumulation of acetic and butyric acid led to the decreasing of pH (<5.5), and provided stress to the cell, which may cause the cell to die rapidly. With the addition of CaCO_3_, not only the produced acetic and butyric acid was neutralized from undissociated form into dissociated form, but the medium had an increased buffering capacity, which improved the viability of DG-8052 cells. At the same time, the soluble Ca^2+^ maybe transmembrane and affect to the enzymes activities of some key cellular processes, such as sugar transport, butanol tolerance and solventogenesis. The transcriptional analysis showed multiple genes prominently responded to the supplementary CaCO_3_. These genes are primarily responsible for glycolysis to solvent/acid production, solventogensis and sporulation, cell motility and division, molecular chaperones, where the specific gene target need more experiment verification. And there had 5–8% altered genes with unknown functions. Genome re-sequencing result showed that had no general or specific regulator mutated. It suggests the CaCO_3_ associated response for intracellular metabolisms be significantly pleiotropic. This study enables us to deeply understand the multitude of regulatory function CaCO_3_ has in clostridia, and provides valuable targets for engineered strain development of *Clostridium* species to degeneration prevention.

## Methods

### Bacterial strains

*Clostridium beijerinckii* NCIMB 8052 is a wild-type strain purchased from ATCC (ATCC 51743, WT-8052), and *C. beijerinckii* DG-8052, a degenerate strain of wild type NCIMB 8052, was generated as described previously^13^.

### Growth and fermentation

The stock culture of DG-8052 was maintained at −75 °C, and revived by sequential culture in anoxic sterile tryptone glucose yeast extract (TGY) medium. Seeds cultures were inoculated into sterile TGY medium and incubated for 12 to 14 h at 35 ± 1 °C in anaerobic chamber (Coy Laboratory Products Inc., Ann Arbor, Michigan) with a modified atmosphere of 82% N_2_, 15% CO_2_, and 3% H_2_. For batch fermentation, DG-8052 precultures in TGY medium (6%) were transferred to loosely capped 250 ml Pyrex medium bottles containing semi-defined P2 medium plus 4 g/l CaCO_3_. The DG-8052 culture without CaCO_3_ was used as the control. Unless otherwise stated, all fermentations were conducted in triplicate at 35 ± 1 °C for 72 h without agitation or pH control. The pH profile was monitored with a Beckman Ф500 pH meter (Beckman Coulter Inc., Brea, CA). Growth of *C. beijerinckii* was estimated using the F-7000 spectrophotometer (Hitachi High-Tech Inc., JP) to measure OD_600_. The concentrations of fermentation products (acetate, butyrate, acetone, butanol, ethanol), were measured using a 7890 A Agilent Technologies gas chromatograph (Agilent Technologies Inc., Wilmington, DE) equipped with a flame ionization detector (FID) and 30 m (length) x 320 m (internal diameter) x 0.50 m (HP-Innowax film) J x W 19091N-213 capillary column as described previously^12^.

The cells were collected at 12 h and 24 h for the morphological observation under scanning electron microscope (Hitachi TM-1000, JP). And at the same time, cell pellets were used to purify total RNA for microarray analysis.

### Total RNA purification

Cellular biomass of DG-8052 in P2 medium and P2 medium plus 4 g/l CaCO_3_ were collected at 12 h (acidogenic phase) and 24 h (early solventogenic phase) of fermentation. Total cellular RNA was extracted from homogenized cells using RiboPure^TM^ RNA Purification Kit, bacteria (Ambion^®^, Life Technologies, Inc., US). RNA concentration was measured by NanoDrop 1000 (NanoDrop Technologies, Wilmington, DE, US). RNA quality was analyzed by 1.2% denatured formaldehyde gel electrophoresis. RNA samples for microarray hybridization had 23 S:16 S rRNA ≥ 2:1 and A260/A280 ≥ 1.80.

### Comparative microarray hybridization

Generation of complementary DNA (cDNA) and its subsequent amino-allyl labelling was performed as described. Using Crystal Core^®^ cDNA amplified RNA labelling kit (CapitalBio, Beijing, China), 1 μg of total RNA was processed to generate cDNA and labelled using 100 μM each dATP, dTTP, dGTP and 25 μM Cy3-label or Cy5-label dCTP. For two–colour microarray hybridization, Cy5 labelled DG-8052 (P2 with CaCO_3_) cDNA and Cy3 labelled DG-8052 (P2) cDNA from the samples collected at the same time point were pooled resulting to six samples for hybridization (triplicate 12-h samples in triplicate and 24-h samples in triplicate). The microarray probes were designed with Agilent eArray software (https://earray.chem.agilent.com/earray/) based on the genomic sequence of *C. beijerinckii* NCIMB 8052. Out of 5243 annotated genes, 5168 genes were examined capturing 98.6% of the genome. The hybridization was performed by CapitalBio and Agilent Technologies (CapitalBio, Beijing, China) using a custom-made Agilent chip (15000 probes/array). To reduce technique variation, the triplicates of each *C. beijerinckii* 8052 probe sequence (60mer) together with 60 probes of negative and positive control were fabricated on each array. The samples were hybridized to six arrays and the hybridized slides were scanned using Agilent G2565CA Microarray Scanner, and scanned images were extracted and analyzed with GenePix^®^ Pro 7 Microarray Acquisition and Analysis Software (Molecular Devices, Sunnyvale, US).

### Microarray data analysis

Data generated from microarray experiments were processed using Molecular Annotation System V4.0 (CapitalBio, Beijing, China). Firstly, LOWESS (Locally Weighted Scatterplot Smoothing) intensity-based normalization was applied. For each array, gene expression ratio was calculated as dividing the Cy5 intensity (signal of DG-8052 fermented in P2 medium with CaCO_3_) by the Cy3 intensity (signal of DG-8052 fermented in P2 medium). Expression ratio for each gene was the average ratio of replicate. To facilitate a fair comparison of up- and down-regulated genes, fold change was calculated as follows: for genes with an expression ratio ≥1, the fold change is the same as the expression ratio, whereas folds change of genes whose ratio is <1 equals the reciprocal of the expression ratio multiplied by −1.

The data were analyzed by pairwise and point-by-point comparison between the gene expression of DG-8052 in P2 medium with and without CaCO_3_ using SAM (Significant Analysis of Microarrays, version 2.23b). Genes exhibiting expression changes of >2-fold and a p-value that was <0.05 were considered to be significantly altered. Enrichment analysis of Gene Ontology terms (GO), including biological process, cellular component and molecular function, and Kyoto Encyclopedia of Genes and Genomes (KEGG) enrichment pathway analysis were performed using DAVID Functional Annotation Bioinformatics Microarray Analysis (DAVID Bioinformatics Resources 6.7) to identify statistically overrepresented biological terms^18^,^40^,^41^.

### Microarray data accession number

All protocols related to this microarray platform, which include information on probe sequences and synthesis, labelling, hybridization and scan protocols, and microarray data have been submitted to NCBI’s Gene Expression Omnibus database at http://www.ncbi.nlm.nih.gov/geo/ with GEO accession number of GSE81808.

### Real-time quantitative PCR (qRT-PCR)

Following microarray analysis results, 10 up-regulated genes (Cbei_0311, Cbei_2826, Cbei_0795, Cbei_0870, Cbei_1113, Cbei_3472, Cbei_1108, Cbei_1255, Cbei_4644, Cbei_1130) and 10 down-regulated genes (Cbei_4123, Cbei_0331, Cbei_4871, Cbei_1221, Cbei_4669, Cbei_0411, Cbei_2261, Cbei_1227, Cbei_4406, Cbei_4491) were selected to validate microarray by q-RT-PCR. Culture samples for RNA isolation were taken as described before. RNA was isolated and RNA content was determined as described above. For qRT-PCR, primers specific to the 20 genes were designed (Additional file Table S1). These gene-specific primers were synthesized by Sangon Biotech (Shanghai, China). The 16 S rRNA of *C. beijerinckii* 8052 was selected as amplified internal standard for the qRT-PCR analysis. Prior to selection of 16 S rRNA as an internal standard, the expression of 16 s rRNA of CaCO_3_ treated and untreated *C. beijerinckii* DG-8052 cultures was analyzed and confirmed for constant expression under the reaction condition of the study. Total RNAs (2 μg) were reverse transcribed to cDNA with the SuperScript^TM^ III reverse transcriptase (Invitrogen corporation, Carlsbad, CA). The mRNA levels of genes of interest (Additional Table S1) were quantified by subjecting cDNA to Q-RT-PCR analysis in triplicate using a Bio-Rad iCycler continuous fluorescence detection system (Bio-Rad, Hercules, CA). The conditions were as follows: step 1, 95 °C for 2 min, step 2, 95 °C for 15 sec, step 3, 55 °C for 30 sec, 40 cycles of step 2 and 3, step 4, 95 °C for 1 min, step 5, 55 °C for 1 min, and step 6, heat from 65 °C to 95 °C with a ramp speed of 1 °C per 10 sec, resulting in melting curves. Expression levels of *C. beijerinckii* 8052 genes were quantified by the comparative C_T_ method as previously described^12^.

### Genome resequencing

DG-8052 genomic DNA was extracted using Bacterial Genomic DNa Isolation kit (B518225, Sangon Biotech, Shanghai, China) from 50 ml 12 h culture grown in P2 medium. Genomic DNA was firstly fragmented, then adapters were ligated to the ends of fragments, and adapter-modified DNA fragments were enriched by PCR and sequenced by Illumina Hiseq 2500. Finally the filtered reads were aligned to the *Clostridium beijerinckii* NCIMB 8052 reference sequence (GenBank: NC_009617.1). This genomic DNA sequencing was analyzed by the commercial sequencing company RealGene Bio-Tec (Shanghai, China), and the sequences obtained in this study have been submitted to the Sequence Read Archive (SRA) database with accession numbers of SRP082285.

### Statistical analysis

The data were analyzed by ANOVA, and p < 0.05 was selected prior to the experiments to reflect statistical significance. Unless otherwise stated, all results are expressed as the mean ± SD (n ≥ 3). All the analyses were conducted using the General Linear Model (GLM) procedure of SAS Version 9.1.3 (SAS Institute Inc., Cary, NC, USA).

## Additional Information

**How to cite this article**: Jiao, S. *et al*. Transcriptional analysis of degenerate strain *Clostridium beijerinckii* DG-8052 reveals a pleiotropic response to CaCO_3_-associated recovery of solvent production. *Sci. Rep.* **6**, 38818; doi: 10.1038/srep38818 (2016).

**Publisher's note:** Springer Nature remains neutral with regard to jurisdictional claims in published maps and institutional affiliations.

## Supplementary Material

Supplementary Information

## Figures and Tables

**Table 1:** KEGG pathway showing significant changes in *C. beijerinckii* DG-8052 cultures with addition of 4 g/L CaCO_3._

culture	KEGG ID	Pathway definition	*p* value	Benjamini*	Count**
Up-regulated					
12 h	cbe02010	ABC transporters	1.8E-3	1.0E-1	30
	cbe00562	Inositol phosphate metabolism	2.4E-3	7.2E-2	5
	cbe00400	Phenylalanine, tyrosine and tryptophan biosynthesis	2.5E-3	5.0E-2	9
	cbe02020	Two-component system	4.2E-2	6.2E-2	16
	cbe00620	Pyruvate metabolism	4.0E-2	3.9E-2	10
24 h	cbe00040	Pentose and glucuronate interconversions	9.4E-8	6.3E-6	20
	cbe02020	Two-component system	5.6E-4	1.9E-2	24
	cbe00400	Phenylalanine, tyrosine and tryptophan biosynthesis	1.0E-3	2.3E-2	12
	cbe00030	Pentose phosphate pathway	8.1E-3	1.3E-1	16
	cbe00450	Selenoamino acid metabolism	9.1E-3	1.1E-1	8
	cbe02010	ABC transporters	1.0E-2	1.1E-1	40
	cbe00562	Inositol phosphate metabolism	1.5E-2	1.3E-1	5
	cbe00290	Valine, Leucine and isoleucine biosynthesis	3.4E-2	2.5E-1	9
	cbe00480	Glutathione metabolism	6.6E-2	3.1E-1	5
	cbe00620	Pyruvate metabolism	6.6E-2	3.7E-1	13
	cbe00020	Citrate cycle (TCA cycle)	7.6E-2	3.8E-1	5
Down-regulated					
12 h	cbe00190	Oxidative phosphorylation	5.2E-4	2.8E-2	10
	cbe00330	Arginine and proline metabolism	7.5E-4	2.0E-2	11
	cbe00440	Phosphonate and phosphinate metabolism	1.1E-2	1.8E-1	4
	cbe00250	Alanine, aspartate and glutamate metabolism	6.5E-2	6.0E-1	7
	cbe00230	Purine metabolism	9.0E-2	6.5E-1	11
24 h	cbe00440	Phosphonate and phosphinate metabolism	4.0E-3	1.3E-1	5
	cbe00730	Thiamine metabolism	1.7E-3	1.1E-1	9
	cbe00860	Porphyrin and chlorophyll metabolism	6.3E-3	1.4E-1	11
	cbe00330	Arginine and proline metabolism	2.6E-2	3.7E-1	11
	cbe00190	Oxidative phosphorylation	4.5E-2	4.7E-1	9

^*^Significant groups were selected based upon Benjamini (<0.05).

^**^Number of genes within the given KEGG ID showing a significant change in their expression level.

**Table 2:**
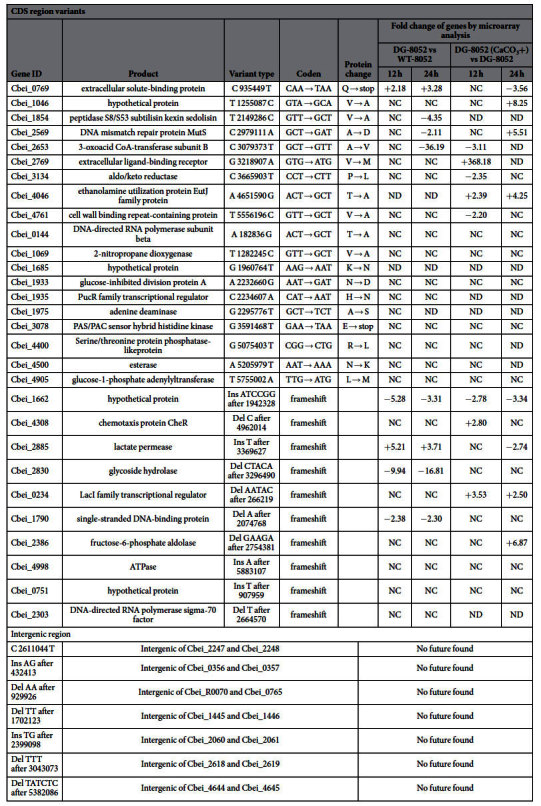
Variants identified in *Clostridium beijerinckii* DG-8052^a^ and their transcriptional change during fermentation.

^a^single nucleotide variations (SNVs) and InDel were identified by aligning the sequenced data with the reference genome of *C. beijerinckii* NCIMB8052.

^*^ND, not detected, NC, no changed.
