# Supplementary material for: Transcriptional analysis of degenerate strain Clostridium beijerinckii DG-8052 reveals a pleiotropic response to CaCO3-associated recovery of solvent production
Source: Sci Rep. 2016 Dec 14;6:38818. doi: 10.1038/srep38818 (PMC5155275; doi:10.1038/srep38818)
Supplement: Supplementary Information [file srep38818-s1.pdf]

Transcriptional analysis of degenerate strain *Clostridium beijerinckii* DG-8052 reveals a pleiotropic response to $CaCO_3$-associated recovery of solvent production

Shengyin Jiao[1], Yan Zhang[2], Caixia Wan[3], Jia Lv[1], Renjia Du[1], Ruijuan Zhang[1], Bei Han[1*]

[1]School of Public Health, Health Science Center, Xi'an Jiaotong University, Xi'an, China

[2]Institute for Genome Sciences, University of Maryland, School of Medicine, Baltimore, Maryland, US

[3]Department of Bioengineering, University of Missouri Columbia, Missouri, US

Shengyin Jiao and Yan Zhang contribute equally to this paper.

*Corresponding author

Mailing address: P.O.44, NO.76 Yanta West Road, School of Public Health, Health Science Center, Xi'an Jiaotong University, Xi'an, 710061, Shaanxi, China.

Additional files

FigS1. q-RT-PCR analysis results to show the mRNA levels changes of selected genes in *C. beijerinckii* DG-8052 in P2 medium with $CaCO_3$ at 12h (A) and 24 h (B). heat shock protein Hsp20 (Hsp20, Cbei_4123), inosine 5'-monophosphate dehydrogenase (IMPDH, Cbei_0331), transketolase domain-containing protein (TK, Cbei_4871), adenylosuccinate lyase (ADSL, Cbei_1221), glycoside hydrolase (GH, Cbei_4669), electron transfer flavo protein subunit alpha-like protein(Alpha-ETF, Cbei_0311), carbohydrate-binding family V/XII protein (CBD V/XII, Cbei_2826), 4Fe-4S ferredoxin (FDX, Cbei_0795), hypothetical protein(H-P, Cbei_0870), peptidase U32 (P-U32); 24h: acetyl-CoA acetyltransferase (ACAT, Cbei_0411), lytic transglycosylase (LTs, Cbei_2261), 6,7-dimethyl-8-ribityllumazine synthase (RITS, Cbei_1227), hypothetical protein (H-P1, Cbei_4406), thiW protein (ThiW, Cbei_4491), oxidoreductase FAD/NAD(P)-binding subunit (OR, Cbei_3472), ferric uptake regulator family protein (FUR, Cbei_1108), hypothetical protein (H-P2, Cbei_1255), transketolase (TKTL, Cbei_4644), phosphate ABC transporter ATPase (ATPase, Cbei_1130).

Table S1. List of 20 genes and sequences of primers used in Q-RT-PCR experiments for the validation of microarray analysis

Table S2 COG functional classification showing significant changes in *C. beijerinckii* DG-8052 cultures with addition of 4g/L $CaCO_3$

Table S3A. Differentially expressed (up regulation) genes in DG-8052 cultures with addition of 4g/L $CaCO_3$ vs DG-8052 during acidogenic phase

Table S3B. Differentially expressed (down regulation) genes in DG-8052 cultures with addition of 4g/L $CaCO_3$ vs DG-8052 during acidogenic phase

Table S3C. Differentially expressed (up regulation) genes in DG-8052 cultures with addition of 4g/L $CaCO_3$ vs DG-8052 during solventogenic phase

Table S3D. Differentially expressed (down regulation) genes in DG-8052 cultures with addition of 4g/L $CaCO_3$ vs DG-8052 during solventogenic phase

Table S4A. Genes differentially expressed by more than 5 folds in DG-8052 cultures with addition of 4g/L $CaCO_3$ vs DG-8052 during acidogenic phase

Table S4B. Genes differentially expressed by more than 5 folds in DG-8052 cultures with addition of 4g/L $CaCO_3$ vs DG-8052 during solventogenic phase

Table S5A. Fold change of solvent production genes in DG-8052 cultures with addition of 4g/L $CaCO_3$ vs DG-8052 by microarray analysis

Table S5B. Fold change of sugar transporter genes in DG-8052 cultures with addition of 4g/L $CaCO_3$ vs DG-8052 as determined by microarray analysis

Table S5C. Fold change of amino acids biosynthesis / metabolism genes in DG-8052 cultures with addition of 4g/L $CaCO_3$ vs DG-8052 according to microarray analysis.

Table S5D. Fold change of sigma factor & sporulation genes in DG-8052 cultures with addition of 4g/L $CaCO_3$ vs DG-8052 according to microarray analysis.

Table S5E. Fold change of cell division proteins in DG-8052 cultures with addition of 4g/L $CaCO_3$ vs DG-8052 according to microarray analysis.

Fig. S1.

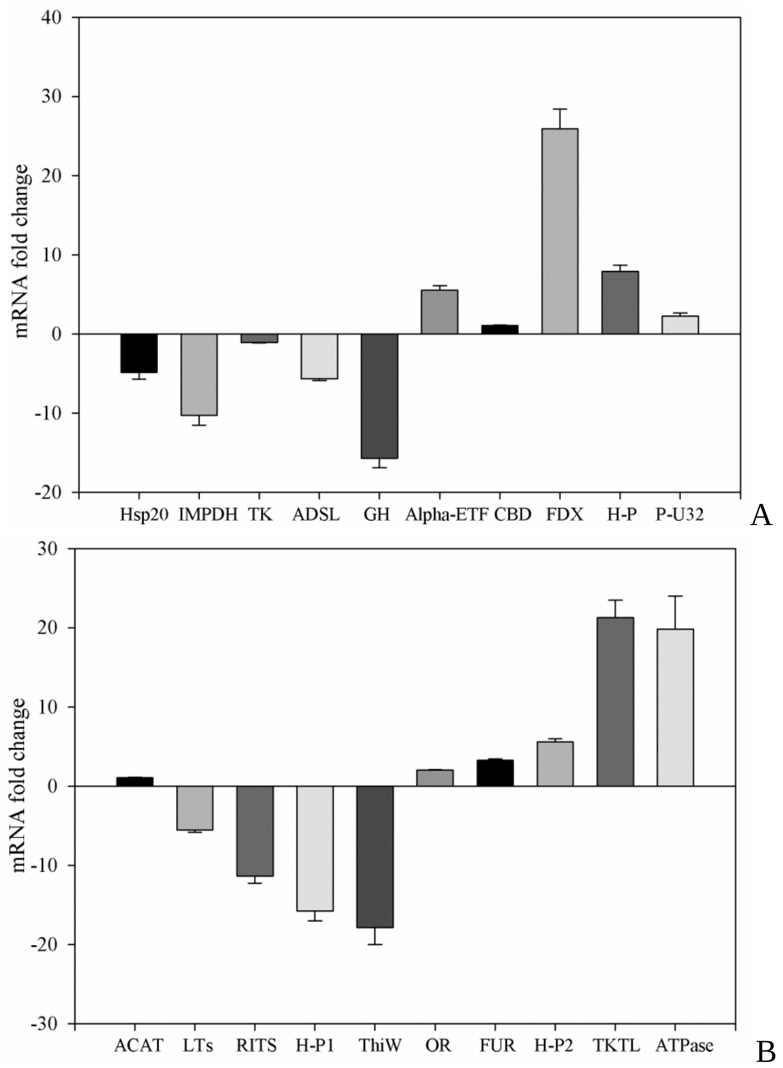
40
30
20
10
0
-10
-20
mRNA fold change
Hsp20
IMPDH
TK
ADSL
GH
Alpha-ETF
CBD
FDX
H-P
P-U32
A
30
20
10
0
-10
-20
-30
mRNA fold change
ACAT
LTs
RITS
H-P1
ThiW
OR
FUR
H-P2
TKTL
ATPase
B

Table S1. List of 20 genes and sequences of primers used in validation of microarray analysis by Q-RT-PCR

Gene symbol	Protein	Fold change DG-8052 in P2+$CaCO_3$ vs DG-8052 in P2 medium		primer sequence	Length (bp)
		Microarray analysis	Q-RT-PCR		
Cbei_4123	heat shock protein Hsp20	-3.15*	-4.85*	F: 5′ AACCCAAACTCATTGGTGAA 3′<br>R: 5′ ATGTTTGGATTGATACCTTT 3′	115
Cbei_0331	inosine 5'-monophosphate dehydrogenase	-8.48*	-10.3*	F: 5′ GAAGGCCGTCTAAAGGGATT 3′<br>R: 5′ CACCTACTGCTGCTCCACAT 3′	121
Cbei_4871	transketolase domain-containing protein	-2.36*	-1.09*	F: 5′ CAGACATTGATGCTTCCCAA 3′<br>R: 5′ AATGGCATTAGCAGGAAAGC 3′	129
Cbei_1221	adenylosuccinate lyase	-3.25*	-5.67*	F: 5′ CGGAAATTGCTCAAAGTGC 3′<br>R: 5′ TGCCATAGCTGATGATCCTATT 3′	113
Cbei_4669	glycoside hydrolase	-11.1*	-15.7*	F:5’ TTGGTGATGAAGAGCTTGAAA 3’<br>R:5’ TGGCTTACATTCAACGAAATAAA 3’	150
Cbei_0411	acetyl-CoA acetyltransferase	-2.73**	+1.08**	F: 5′ TGTGGATCAGGTCTTAGAGCA 3′<br>R: 5′ CCCATCTTGCTTTGTCCAAT 3′	130
Cbei_2261	lytic transglycosylase	-3.98**	-5.54**	F: 5’ CCAGCAAAACTTGCGAATCT 3’<br>R: 5’ CCATCACCAGATGAGGGACT 3’	197
Cbei_1227	6,7-dimethyl-8-ribityllumazine synthase	-6.04**	-11.37**	F:5’ CTCTTGAAAGTGGAAAGCCA 3’<br>R: 5’ CGCCATTTCTATTGCTGAAA 3’	131
Cbei_4406	hypothetical protein	-10.8**	-15.78**	F: 5’ CCAATAAACTTCCTGTTCCCA 3’<br>R: 5’ TTCAATACCGATTGGAGCTG 3’	149
Cbei_4491	thiW protein	-11.1**	-17.89**	F: 5’ TCTCCAATTACTGCAATGCC 3’<br>R: 5’ GAATGCATTTGTGACATCCC 3’	141
Cbei_0311	electron transfer flavo protein subunit alpha-like protein	+3.25*	+5.51*	F: 5′ CGGTAGAACTGTAAGGCCAAG 3′<br>R: 5′ TGGTGCTTTATCATCAGCATT 3′	135

Cbei_2826	carbohydrate-binding family V/XII protein	+2.81*	+1.10*	F: 5’ TCAATGGTGGAACAGTTGGA 3’<br>R: 5’ AGCGTCTTAGCAGGTTGTCC 3’	319
Cbei_0795	4Fe-4S ferredoxin	+28.6*	+25.9*	F:5’ AAACAATTCAATTTGGATGCC 3’<br>R: 5’ TTTAATTTGCACAGGCCATT 3’	128
Cbei_0870	hypothetical protein	+5.32*	+7.89*	F:5’ GACACCATAGAAGGAATTACGGA 3’<br>R:5’TCCAGGATTTATATCTAATACAACACG 3’	99
Cbei_1113	peptidase U32	+4.5*	+2.25*	F:5’ AATTAAAGGAAGGCGTGGAA3’<br>R: 5’ TTGCATCAACACCTGTTTCA 3’	138
Cbei_3472	oxidoreductase FAD/NAD(P)-binding subunit	+3.94**	+2.01**	F: 5’ CCTGATTGAGCAAAGCTCCT 3’<br>R: 5’ GTTGTCTCAGGACCTCATGG 3’	110
Cbei_1108	ferric uptake regulator family protein	+5.15**	+3.25**	F: 5’ CAAATCCATTTCCAATGTCG 3’<br>R: 5’ TTAGTCTCGTCCCAAAGCG 3’	133
Cbei_1255	hypothetical protein	+9.03**	+5.59**	F: 5’ CATATTCCGGCAGGTGTTT 3’<br>R: 5’ CGCTCCTTTAAGCTTTGCAT 3’	132
Cbei_4644	transketolase	+11.0**	+21.28**	F:5’ CTGCTTTACCTGATGCTCCA 3’<br>R: 5’ GCGCAAGATTAGCAGTTGAA 3’	108
Cbei_1130	phosphate ABC transporter ATPase	+14.8**	+19.79**	F: 5’ CTTAAGAGGAGCAGCGCTTT 3’<br>R: 5’ GCAGATGTTGGTTCATCCAT 3’	150

*Fold change at 12h culture of *C. beijerinckii* DG8052 in P2+$CaCO_3$ medium; ** Fold change at 24h culture of *C. beijerinckii* DG8052 in P2+$CaCO_3$ medium.

Table S2 COG functional classification showing significant changes in *C.beijerinckii* DG-8052 cultures with addition of 4g/L $CaCO_3$

culture	COG	COG definition	*p* value	Benjamini[*]	Count[**]
Up-regulated					
12h	E	Amino acid transport and metabolism	1.40E-04	4.70E-03	19
24h	T	Signal transduction mechanisms / Transcription	1.20E-02	2.90E-01	12
	J	Translation, ribosomal structure and biogenesis	8.60E-02	7.20E-01	8
Down-regulated					
12h	E	Amino acid transport and metabolism	6.20E-03	1.90E-01	21
	V	Defense mechanisms	4.70E-02	5.60E-01	19
24h	J	Translation, ribosomal structure and biogenesis	2.60E-02	5.50E-01	11

*significant groups were selected based upon Benjamini (<0.05);

**number of genes within the given KEGG ID showing a significant change in their expression level;

Table S3A. Differentially expressed (up-regulation) genes in DG-8052 in P2 medium with $CaCO_3$ vs DG-8052 in P2 medium during acidogensis

GOTERM_ Category	GO number	Term	Count	%	P-value
Biological Process					
	0043648	dicarboxylic acid metabolic process	11	2.01	5.44E-04
	0009072	aromatic amino acid family metabolic process	9	1.65	0.001
	0046417	chorismate metabolic process	9	1.65	0.001
	0009073	aromatic amino acid family biosynthetic process	9	1.65	0.001
	0046394	carboxylic acid biosynthetic process	24	4.39	0.002
	0016053	organic acid biosynthetic process	24	4.39	0.002
	0006808	regulation of nitrogen utilization	4	0.73	0.004
	0008652	cellular amino acid biosynthetic process	20	3.66	0.004
	0009309	amine biosynthetic process	20	3.66	0.007
	0046174	polyol catabolic process	4	0.73	0.009
	0042493	response to drug	8	1.46	0.025
	0015893	drug transport	8	1.46	0.025
	0015837	amine transport	7	1.28	0.026
	0006020	inositol metabolic process	3	0.55	0.030
	0009095	aromatic amino acid family biosynthetic process, prephenate pathway	3	0.55	0.030
	0019310	inositol catabolic process	3	0.55	0.030
	0007610	behavior	15	2.75	0.039
	0006935	chemotaxis	15	2.75	0.039
	0007626	locomotory behavior	15	2.74	0.039
	0042330	taxis	15	2.74	0.039
	0015849	organic acid transport	8	1.46	0.041
	0046942	carboxylic acid transport	8	1.46	0.041
	0015850	organic alcohol transport	5	0.91	0.049
	0015904	tetracycline transport	5	0.91	0.049
	0042891	antibiotic transport	5	0.91	0.049
	0055114	oxidation reduction	30	5.48	0.052
Cellular Component					
	0044462	external encapsulating structure part	8	1.46	0.024
	0030288	outer membrane-bounded periplasmic space	7	1.28	0.036
	0042597	periplasmic space	7	1.28	0.047

	0030313	cell envelope	11	2.01	0.054
Molecular Function					
	0016887	ATPase activity	29	5.30	0.025
	0015293	symporter activity	19	3.47	0.006
	0015294	solute:cation symporter activity	19	3.47	0.006
	0042626	ATPase activity, coupled to transmembrane movement of substances	14	2.56	0.009
	0043492	ATPase activity, coupled to movement of substances	14	2.56	0.009
	0051119	sugar transmembrane transporter activity	19	3.47	0.011
	0016628	oxidoreductase activity, acting on the CH-CH group of donors, NAD or NADP as acceptor	5	0.91	0.011
	0016820	hydrolase activity, acting on acid anhydrides, catalyzing transmembrane movement of substances	14	2.56	0.012
	0005402	cation:sugar symporter activity	17	3.11	0.015
	0015399	primary active transmembrane transporter activity	14	2.56	0.021
	0015405	P-P-bond-hydrolysis-driven transmembrane transporter activity	14	2.56	0.021
	0042623	ATPase activity, coupled	17	3.11	0.027
	0015238	drug transporter activity	8	1.46	0.030
	0008509	anion transmembrane transporter activity	7	1.28	0.030
	0016769	transferase activity, transferring nitrogenous groups	9	1.65	0.033
	0008483	transaminase activity	8	1.46	0.036
	0015295	solute:hydrogen symporter activity	15	2.74	0.051
	0005351	sugar:hydrogen symporter activity	15	2.74	0.051

Table S3B. Differentially expressed (down-regulation) genes in DG-8052 in P2 medium with $CaCO_3$ vs DG-8052 in P2 medium during acidogensis

GOTERM _Category	GO number	Term	Count	%	P-value
Biological Process					
	0034220	ion transmembrane transport	10	1.52	4.49E-06
	0015985	energy coupled proton transport, down electrochemical gradient	8	1.21	2.56E-05
	0015986	ATP synthesis coupled proton transport	8	1.21	2.56E-05
	0015992	proton transport	8	1.21	6.33E-05
	0006818	hydrogen transport	8	1.21	6.33E-05
	0015672	monovalent inorganic cation transport	11	1.67	1.88E-04
	0006119	oxidative phosphorylation	8	1.21	8.11E-04
	0009142	nucleoside triphosphate biosynthetic process	11	1.67	8.18E-04
	0044271	nitrogen compound biosynthetic process	46	6.97	9.39E-04
	0009165	nucleotide biosynthetic process	18	2.73	0.001
	0046034	ATP metabolic process	10	1.52	0.001
	0006754	ATP biosynthetic process	10	1.52	0.001
	0006811	ion transport	21	3.18	0.001
	0006164	purine nucleotide biosynthetic process	13	1.97	0.001
	0009141	nucleoside triphosphate metabolic process	11	1.67	0.002
	0009206	purine ribonucleoside triphosphate biosynthetic process	10	1.52	0.002
	0009145	purine nucleoside triphosphate biosynthetic process	10	1.52	0.002
	0009201	ribonucleoside triphosphate biosynthetic process	10	1.52	0.002
	0006163	purine nucleotide metabolic process	13	1.97	0.002
	0006812	cation transport	17	2.58	0.003
	0009199	ribonucleoside triphosphate metabolic process	10	1.52	0.003
	0009205	purine ribonucleoside triphosphate metabolic process	10	1.52	0.003
	0009144	purine nucleoside triphosphate metabolic process	10	1.52	0.003
	0009064	glutamine family amino acid metabolic process	8	1.21	0.032
	0006526	arginine biosynthetic process	4	0.60	0.039
Cellular Component					

	0045259	proton-transporting ATP synthase complex	8	1.21	1.10E-07
	0016469	proton-transporting two-sector ATPase complex	8	1.21	1.46E-06
	0045261	proton-transporting ATP synthase complex, catalytic core F(1)	5	0.76	1.76E-04
	0033178	proton-transporting two-sector ATPase complex, catalytic domain	5	0.76	0.001
	0006793	phosphorus metabolic process	26	3.94	0.003
	0009152	purine ribonucleotide biosynthetic process	11	1.67	0.004
	0034654	nucleobase, nucleoside, nucleotide and nucleic acid biosynthetic process	19	2.88	0.005
	0034404	nucleobase, nucleoside and nucleotide biosynthetic process	19	2.88	0.005
	0009150	purine ribonucleotide metabolic process	11	1.67	0.005
	0042777	plasma membrane ATP synthesis coupled proton transport	4	0.61	0.006
	0006350	transcription	49	7.42	0.006
	0006796	phosphate metabolic process	25	3.79	0.006
	0009253	peptidoglycan catabolic process	7	1.06	0.007
	0006027	glycosaminoglycan catabolic process	7	1.06	0.007
	0009260	ribonucleotide biosynthetic process	11	1.67	0.010105
	0016310	phosphorylation	23	3.48	0.011
	0009259	ribonucleotide metabolic process	11	1.67	0.012
	0006026	aminoglycan catabolic process	7	1.06	0.014
	0055085	transmembrane transport	10	1.52	0.014
	0006525	arginine metabolic process	5	0.76	0.015
	0033177	proton-transporting two-sector ATPase complex, proton-transporting domain	3	0.45	0.018
	0045263	proton-transporting ATP synthase complex, coupling factor F(o)	3	0.45	0.018
Molecular Function					
	0046933	hydrogen ion transporting ATP synthase activity, rotational mechanism	7	1.06	8.16E-05
	0015078	hydrogen ion transmembrane transporter activity	8	1.21	9.38E-05
	0015077	monovalent inorganic cation transmembrane transporter activity	9	1.36	1.58E-04
	0022890	inorganic cation transmembrane transporter activity	12	1.82	0.003
	0046961	proton-transporting ATPase activity, rotational mechanism	5	0.76	0.003
	0003796	lysozyme activity	5	0.76	0.011
	0019829	cation-transporting ATPase activity	5	0.76	0.011

	0019898	extrinsic to membrane	4	0.61	0.014
	0042625	ATPase activity, coupled to transmembrane movement of ions	8	1.21	0.043

Table S3C. Differentially expressed (up-regulation) genes in DG-8052 in P2 medium with $CaCO_3$ vs DG-8052 in P2 medium during solventogenesis

GOTERM_ Category	GO number	Term	Count	%	P-value
Biological Process					
	0015893	drug transport	14	1.56	0.001
	0042493	response to drug	14	1.56	0.001
	0006820	anion transport	11	1.22	0.002
	0009073	aromatic amino acid family biosynthetic process	11	1.22	0.004
	0046417	chorismate metabolic process	11	1.22	0.004
	0009072	aromatic amino acid family metabolic process	11	1.22	0.004
	0015698	inorganic anion transport	9	1.00	0.005
	0042891	antibiotic transport	8	0.89	0.008
	0015850	organic alcohol transport	8	0.89	0.008
	0015904	tetracycline transport	8	0.89	0.008
	0042430	indole and derivative metabolic process	5	0.56	0.014
	0046219	indolalkylamine biosynthetic process	5	0.56	0.014
	0000162	tryptophan biosynthetic process	5	0.56	0.014
	0006568	tryptophan metabolic process	5	0.56	0.014
	0042434	indole derivative metabolic process	5	0.56	0.014
	0008272	sulfate transport	5	0.56	0.014
	0042435	indole derivative biosynthetic process	5	0.56	0.014
	0006586	indolalkylamine metabolic process	5	0.56	0.014
	0005996	monosaccharide metabolic process	24	2.67	0.014
	0043648	dicarboxylic acid metabolic process	12	1.34	0.016
	0009082	branched chain family amino acid biosynthetic process	7	0.78	0.022
	0009081	branched chain family amino acid metabolic process	7	0.78	0.022
	0010035	response to inorganic substance	4	0.45	0.023
	0006808	regulation of nitrogen utilization	4	0.45	0.023
	0006979	response to oxidative stress	4	0.45	0.023
	0006534	cysteine metabolic process	5	0.56	0.027
	0046677	response to antibiotic	9	1.00	0.031
	0055114	oxidation reduction	50	5.57	0.044
	0042330	taxis	23	2.56	0.045
	0007626	locomotory behavior	23	2.56	0.045
	0007610	behavior	23	2.56	0.045
	0006935	chemotaxis	23	2.56	0.045
Molecular Function					
	0016744	transferase activity, transferring aldehyde or ketonic groups	10	1.11	2.64E-04
	0015238	drug transporter activity	14	1.56	0.001

	0015297	antiporter activity	15	1.67	0.002
	0015103	inorganic anion transmembrane transporter activity	9	1.00	0.003
	0016836	hydro-lyase activity	13	1.45	0.003
	0016860	intramolecular oxidoreductase activity	12	1.34	0.004
	0016861	intramolecular oxidoreductase activity, interconverting aldoses and ketoses	11	1.23	0.004
	0015299	solute:hydrogen antiporter activity	9	1.00	0.005
	0015298	solute:cation antiporter activity	9	1.00	0.005
	0015300	solute:solute antiporter activity	9	1.00	0.005
	0015520	tetracycline:hydrogen antiporter activity	8	0.89	0.009
	0016209	antioxidant activity	8	0.89	0.009
	0008493	tetracycline transporter activity	8	0.89	0.009
	0015307	drug:hydrogen antiporter activity	8	0.89	0.009
	0042895	antibiotic transporter activity	8	0.89	0.009
	0008565	protein transporter activity	11	1.22	0.013
	0008509	anion transmembrane transporter activity	10	1.11	0.021
	0019842	vitamin binding	21	2.34	0.022
	0004802	transketolase activity	4	0.45	0.024
	0043492	ATPase activity, coupled to movement of substances	19	2.12	0.028
	0042626	ATPase activity, coupled to transmembrane movement of substances	19	2.12	0.028
	0016820	hydrolase activity, acting on acid anhydrides, catalyzing transmembrane movement of substances	19	2.12	0.038
	0030976	thiamin pyrophosphate binding	6	0.67	0.043
Cellular Component					
	0031224	intrinsic to membrane	111	12.37	5.77E-05
	0016021	integral to membrane	109	12.14	2.37E-04
	0030313	cell envelope	15	1.67	0.021
	0031975	envelope	15	1.67	0.026
	0030312	external encapsulating structure	15	1.67	0.040

Table S3D. Differentially expressed (down-regulation) genes in DG-8052 in P2 medium with $CaCO_3$ vs DG-8052 in P2 medium during solventogenesis.

GOTERM_ Category	GO number	Term	Count	%	P-value
Biological Process					
	0015985	energy coupled proton transport, down electrochemical gradient	8	0.87	4.05E-04
	0015986	ATP synthesis coupled proton transport	8	0.87	4.05E-04
	0015992	proton transport	8	0.87	9.43E-04
	0006818	hydrogen transport	8	0.87	9.43E-04
	0015672	monovalent inorganic cation transport	12	1.30	0.001
	0042364	water-soluble vitamin biosynthetic process	20	2.17	0.002
	0006767	water-soluble vitamin metabolic process	20	2.17	0.002
	0006754	ATP biosynthetic process	12	1.30	0.002
	0046034	ATP metabolic process	12	1.30	0.002
	0044271	nitrogen compound biosynthetic process	62	6.73	0.002
	0009119	ribonucleoside metabolic process	11	1.19	0.002
	0018130	heterocycle biosynthetic process	29	3.15	0.003
	0009201	ribonucleoside triphosphate biosynthetic process	12	1.30	0.003
	0009206	purine ribonucleoside triphosphate biosynthetic process	12	1.30	0.003
	0009145	purine nucleoside triphosphate biosynthetic process	12	1.30	0.003
	0006766	vitamin metabolic process	20	2.17	0.003
	0009110	vitamin biosynthetic process	20	2.17	0.003
	0009141	nucleoside triphosphate metabolic process	13	1.41	0.003
	0009205	purine ribonucleoside triphosphate metabolic process	12	1.30	0.004
	0009199	ribonucleoside triphosphate metabolic process	12	1.30	0.004
	0009144	purine nucleoside triphosphate metabolic process	12	1.30	0.004
	0046128	purine ribonucleoside metabolic process	9	0.98	0.005
	0034220	ion transmembrane transport	8	0.87	0.006
	0009142	nucleoside triphosphate biosynthetic process	12	1.30	0.006
	0042278	purine nucleoside metabolic process	9	0.98	0.008
	0006119	oxidative phosphorylation	8	0.87	0.010
	0033014	tetrapyrrole biosynthetic process	13	1.41	0.011
	0033013	tetrapyrrole metabolic process	13	1.41	0.011
	0006779	porphyrin biosynthetic process	13	1.41	0.011
	0006778	porphyrin metabolic process	13	1.41	0.011
	0009152	purine ribonucleotide biosynthetic process	13	1.41	0.011

	0001522	pseudouridine synthesis	6	0.65	0.012
	0009150	purine ribonucleotide metabolic process	13	1.41	0.014
	0006812	cation transport	20	2.17	0.015
	0006164	purine nucleotide biosynthetic process	14	1.52	0.018
	0042777	plasma membrane ATP synthesis coupled proton transport	4	0.43	0.020
	0006091	generation of precursor metabolites and energy	17	1.85	0.022
	0006793	phosphorus metabolic process	32	3.47	0.022
	0051186	cofactor metabolic process	28	3.04	0.025
	0006163	purine nucleotide metabolic process	14	1.52	0.027
	0009116	nucleoside metabolic process	13	1.41	0.028
	0009260	ribonucleotide biosynthetic process	13	1.41	0.028
	0009236	cobalamin biosynthetic process	10	1.09	0.030
	0009235	cobalamin metabolic process	10	1.09	0.030
	0009259	ribonucleotide metabolic process	13	1.41	0.034
	0006796	phosphate metabolic process	31	3.37	0.034
	0006811	ion transport	23	2.50	0.038
	0009064	glutamine family amino acid metabolic process	10	1.09	0.039
	0016310	phosphorylation	29	3.15	0.0402
	0034404	nucleobase, nucleoside and nucleotide biosynthetic process	22	2.39	0.042
	0034654	nucleobase, nucleoside, nucleotide and nucleic acid biosynthetic process	22	2.39	0.042
Cellular Component					
	0045259	proton-transporting ATP synthase complex	8	0.87	3.98E-06
	0016469	proton-transporting two-sector ATPase complex	8	0.87	4.76E-05
	0045261	proton-transporting ATP synthase complex, catalytic core F(1)	5	0.54	0.001
	0019861	flagellum	11	1.19	0.005
	0042995	cell projection	11	1.19	0.005
	0033178	proton-transporting two-sector ATPase complex, catalytic domain	5	0.54	0.007
Molecular Function					
	0046933	hydrogen ion transporting ATP synthase activity, rotational mechanism	7	0.76	5.83E-04
	0015078	hydrogen ion transmembrane transporter activity	8	0.87	8.55E-04
	0015077	monovalent inorganic cation transmembrane transporter activity	9	0.98	0.002
	0016866	intramolecular transferase activity	10	1.09	0.004
	0046961	proton-transporting ATPase activity, rotational mechanism	5	0.54	0.010

	0009982	pseudouridine synthase activity	6	0.65	0.011
	0008026	ATP-dependent helicase activity	9	0.98	0.021
	0070035	purine NTP-dependent helicase activity	9	0.98	0.021
	0022890	inorganic cation transmembrane transporter activity	12	1.30	0.032
	0019829	cation-transporting ATPase activity	5	0.54	0.036
	0050660	FAD binding	14	1.52	0.041

Table S4A. Genes differentially expressed by more than 5 folds in DG-8052 in P2 medium with $CaCO_3$ vs DG-8052 in P2 medium during acidogensis.

Fold Change	up/ down	Gene Symbol	Gene Product
4.5	up	Cbei_0238	hypothetical protein
4.5	up	Cbei_3629	hypothetical protein
4.6	up	Cbei_4551	fructose-bisphosphate aldolase
4.7	up	Cbei_5006	LrgB family protein
4.8	up	Cbei_4688	PAS/PAC sensor-containing diguanylate cyclase
4.8	up	Cbei_2306	phage integrase family protein
4.8	up	Cbei_1478	PTS system lactose/cellobiose family transportersubunit IIC
4.8	up	Cbei_5058	EmrB/QacA family drug resistance transporter
4.8	up	Cbei_3364	natural resistance-associated macrophageprotein
4.9	up	Cbei_1205	tRNA pseudouridine synthase B
4.9	up	Cbei_1282	di-trans-poly-cis-decaprenylcistransferase
4.9	up	Cbei_1182	ribonuclease HII
4.9	up	Cbei_4101	secretion protein HlyD family protein
4.9	up	Cbei_4432	monosaccharide-transporting ATPase
4.9	up	Cbei_4098	MarR family transcriptional regulator
4.9	up	Cbei_3703	transport system permease
5	up	Cbei_1482	hypothetical protein
5	up	Cbei_4935	LuxR family transcriptional regulator
5.1	up	Cbei_3625	methyl-accepting chemotaxis sensory transducer
5.1	up	Cbei_1493	cell wall hydrolase/autolysin
5.1	up	Cbei_3811	PTS system lactose/cellobiose-specifictransporter subunit IIA
5.2	up	Cbei_4570	3-dehydroquinate dehydratase
5.2	up	Cbei_4203	glutamate synthase NADH/NADPH small subunit
5.2	up	Cbei_1918	"phosphotransferase system, EIIC"
5.2	up	Cbei_3295	sugar ABC transporter periplasmic protein
5.2	up	Cbei_4217	type II secretion system protein
5.3	up	Cbei_4685	PTS system lactose/cellobiose-specifictransporter subunit IIA
5.3	up	Cbei_1480	LytTR family two component transcriptionalregulator
5.3	up	Cbei_4183	chemotaxis protein CheA
5.3	up	Cbei_0870	hypothetical protein
5.4	up	Cbei_4547	oxidoreductase domain-containing protein
5.4	up	Cbei_3185	methionine--tRNA ligase
5.4	up	Cbei_0270	---
5.4	up	Cbei_4686	sigma-54 factor interaction domain-containingprotein
5.5	up	Cbei_4428	D-galactose-binding periplasmic protein
5.6	up	Cbei_1762	extracellular ligand-binding receptor
5.6	up	Cbei_4980	EmrB/QacA family drug resistance transporter
5.6	up	Cbei_5035	aminoglycoside phosphotransferase
5.7	up	Cbei_4675	glycoside hydrolase
5.7	up	Cbei_4358	AraC family transcriptional regulator
5.7	up	Cbei_2853	major facilitator superfamily transporter
5.7	up	Cbei_5037	dihydrodipicolinate synthase
5.8	up	Cbei_2214	---
5.8	up	Cbei_0235	encapsulation protein CapA

5.9	up	Cbei_1766	ABC transporter
5.9	up	Cbei_5052	hypothetical protein
6	up	Cbei_4006	butyrate kinase
6.1	up	Cbei_4099	EmrB/QacA family drug resistance transporter
6.1	up	Cbei_3518	aldose 1-epimerase
6.1	up	Cbei_4772	methyl-accepting chemotaxis sensory transducer
6.2	up	Cbei_4180	response regulator receiver modulated CheBmethylesterase
6.3	up	Cbei_2771	methyl-accepting chemotaxis sensory transducer
6.3	up	Cbei_1763	inner-membrane translocator
6.4	up	Cbei_4546	xylose isomerase domain-containing protein
6.4	up	Cbei_4078	periplasmic binding protein/LacI transcriptionalregulator
6.4	up	Cbei_0265	hypothetical protein
6.5	up	Cbei_4548	"thiamine pyrophosphate protein, central region"
6.5	up	Cbei_4206	"glutamine synthetase, type I"
6.5	up	Cbei_2213	"peptidase M48, Ste24p"
6.6	up	Cbei_4126	alpha-N-arabinofuranosidase
6.6	up	Cbei_4676	response regulator receiver protein
6.6	up	Cbei_1079	hypothetical protein
6.6	up	Cbei_2793	ABC transporter
6.8	up	Cbei_4543	coenzyme A transferase
7	up	Cbei_1302	ECF subfamily RNA polymerase sigma-24 factor
7	up	Cbei_1481	hypothetical protein
7	up	Cbei_1301	membrane associated protein
7	up	Cbei_2789	L-lactate dehydrogenase
7.1	up	Cbei_4130	type 11 methyltransferase
7.2	up	Cbei_3915	MarR family transcriptional regulator
7.2	up	Cbei_0653	polysaccharide deacetylase
7.3	up	Cbei_4692	TetR family transcriptional regulator
7.3	up	Cbei_3736	major facilitator superfamily transporter
7.3	up	Cbei_0488	hypothetical protein
7.4	up	Cbei_1011	formate acetyltransferase
7.4	up	Cbei_2092	transcriptional regulator TrmB
7.4	up	Cbei_0571	hypothetical protein
7.5	up	Cbei_2061	ammonium transporter
7.5	up	Cbei_4981	MarR family transcriptional regulator
7.6	up	Cbei_0266	hypothetical protein
7.7	up	Cbei_1523	hypothetical protein
7.8	up	Cbei_4205	response regulator receiver/ANTARdomain-containing protein
7.9	up	Cbei_1080	inner-membrane translocator
7.9	up	Cbei_2462	hypothetical protein
7.9	up	Cbei_4097	amino acid permease-associated protein
7.9	up	Cbei_1475	PTS system lactose/cellobiose-specifictransporter subunit IIA
8.1	up	Cbei_1443	Alpha-glucosidase
8.1	up	Cbei_1749	anthranilate synthase component I
8.2	up	Cbei_3299	multidrug ABC transporter ATPase
8.3	up	Cbei_1992	molybdenum ABC transporter periplasmicmolybdate-binding protein
8.3	up	Cbei_1081	ABC transporter

8.3	up	Cbei_1442	binding-protein-dependent transport system innermembrane protein
8.4	up	Cbei_0215	3-isopropylmalate dehydratase small subunit
8.4	up	Cbei_4354	iron-containing alcohol dehydrogenase
8.5	up	Cbei_1906	---
8.5	up	Cbei_2794	ABC transporter
8.6	up	Cbei_2093	multidrug ABC transporter ATPase
8.7	up	Cbei_5033	nitrogen regulatory protein P-II
8.7	up	Cbei_5043	inner-membrane translocator
8.7	up	Cbei_1750	glutamine amidotransferase of anthranilatesynthase
8.8	up	Cbei_1212	"peptidase S14, ClpP"
8.9	up	Cbei_3916	major facilitator superfamily transporter
9	up	Cbei_3591	lysyl-tRNA synthetase
9	up	Cbei_0788	amino acid permease-associated protein
9.1	up	Cbei_2094	multidrug ABC transporter
9.2	up	Cbei_3813	PTS system lactose/cellobiose family transportersubunit IIC
9.2	up	Cbei_4545	sugar transporter
9.5	up	Cbei_0444	glutamine synthetase
9.5	up	Cbei_0214	3-isopropylmalate dehydratase large subunit
9.5	up	Cbei_1951	hypothetical protein
9.7	up	Cbei_4932	binding-protein-dependent transport system innermembrane protein
9.8	up	Cbei_0391	peptidoglycan-binding LysM
9.8	up	Cbei_4221	cell wall binding repeat-containing protein
10	up	Cbei_4433	ABC transporter
10	up	Cbei_5032	metal dependent phosphohydrolase
10	up	Cbei_1303	hypothetical protein
11	up	Cbei_3326	hypothetical protein
11	up	Cbei_2344	hypothetical protein
11	up	Cbei_4349	polar amino acid ABC transporter inner membranesubunit
11	up	Cbei_5059	methyl-accepting chemotaxis sensory transducer
11	up	Cbei_5042	extracellular ligand-binding receptor
12	up	Cbei_1524	sodium:dicarboxylate symporter
12	up	Cbei_0971	CopY family transcriptional regulator
12	up	Cbei_4931	binding-protein-dependent transport system innermembrane protein
12	up	Cbei_4930	peptide ABC transporter ATPase
12	up	Cbei_4928	extracellular solute-binding protein
13	up	Cbei_4693	hypothetical protein
13	up	Cbei_1993	TOBE domain-containing protein
13	up	Cbei_1444	alpha amylase
14	up	Cbei_4346	succinylglutamate desuccinylase/aspartoacylase
14	up	Cbei_4687	phage infection protein
14	up	Cbei_3037	ATP-dependent metalloprotease FtsH
14	up	Cbei_4347	4-aminobutyrate aminotransferase
14	up	Cbei_2097	acyl-protein synthetase LuxE
15	up	Cbei_3036	cell wall binding repeat-containing protein
16	up	Cbei_4929	peptide ABC transporter ATPase
16	up	Cbei_0212	acetolactate synthase small subunit

16	up	Cbei_1949	hypothetical protein
17	up	Cbei_4345	succinylglutamate desuccinylase/aspartoacylase
17	up	Cbei_2343	AsnC family transcriptional regulator
17	up	Cbei_1241	hypothetical protein
18	up	Cbei_0652	tryptophan transport protein
20	up	Cbei_2103	acetyl-CoA hydrolase/transferase
20	up	Cbei_0213	alpha-isopropylmalate/homocitrate synthasefamily transferase
21	up	Cbei_2390	GntR family transcriptional regulator
21	up	Cbei_2758	alanine racemase
27	up	Cbei_3878	extracellular solute-binding protein
29	up	Cbei_0795	4Fe-4S ferredoxin
29	up	Cbei_3876	ribokinase-like domain-containing protein
29	up	Cbei_4352	nitrilase/cyanide hydratase and apolipoproteinN-acyltransferase
32	up	Cbei_2754	"acetyl-CoA carboxylase, biotin carboxylase"
33	up	Cbei_4171	polar amino acid ABC transporter inner membranesubunit
33	up	Cbei_4173	ABC transporter
35	up	Cbei_2011	isopropylmalate/homocitrate/citramalatesynthase-like protein
36	up	Cbei_0794	nicotinate-nucleotide pyrophosphorylase
36	up	Cbei_1939	binding-protein-dependent transport system innermembrane protein
38	up	Cbei_2391	extracellular solute-binding protein
41	up	Cbei_2012	homocitrate synthase
42	up	Cbei_5034	ammonium transporter
42	up	Cbei_2003	nitrogenase molybdenum-iron protein subunitbeta
43	up	Cbei_1598	RpiR family transcriptional regulator
43	up	Cbei_4934	putrescine--2-oxoglutarate aminotransferase
44	up	Cbei_4008	malate dehydrogenase
44	up	Cbei_2342	aspartate ammonia-lyase
47	up	Cbei_0306	ATPase P
48	up	Cbei_2104	beta-lactamase domain-containing protein
48	up	Cbei_4007	2-keto-3-deoxygluconate permease
51	up	Cbei_2001	nitrogen regulatory protein P-II
55	up	Cbei_4170	polar amino acid ABC transporter inner membranesubunit
56	up	Cbei_4172	extracellular solute-binding protein
56	up	Cbei_4350	extracellular solute-binding protein
56	up	Cbei_4351	ABC transporter
64	up	Cbei_1942	dihydropyrimidinase
75	up	Cbei_1998	hypothetical protein
85	up	Cbei_1404	hypothetical protein
105	up	Cbei_1997	hypothetical protein
109	up	Cbei_1936	class III aminotransferase
109	up	Cbei_2000	nitrogen regulatory protein P-II
112	up	Cbei_1941	NMT1/THI5-like protein
115	up	Cbei_2100	vinylacetyl-CoA Delta-isomerase
124	up	Cbei_2757	allophanate hydrolase subunit 1
126	up	Cbei_2755	biotin/lipoyl attachment domain-containingprotein
126	up	Cbei_2763	AsnC family transcriptional regulator
128	up	Cbei_2759	LamB/YcsF family protein

130	up	Cbei_4942	nitrogen regulatory protein P-II
144	up	Cbei_0792	quinolinate synthetase
144	up	Cbei_2761	hypothetical protein
147	up	Cbei_4940	spermidine/putrescine ABC transporter ATPase
158	up	Cbei_4941	extracellular solute-binding protein
160	up	Cbei_1937	iron-containing alcohol dehydrogenase
161	up	Cbei_2006	"ferredoxin, 2Fe-2S"
161	up	Cbei_2767	inner-membrane translocator
165	up	Cbei_4936	aldehyde dehydrogenase
166	up	Cbei_2102	hypothetical protein
166	up	Cbei_1938	ABC transporter
187	up	Cbei_1944	allantoate amidohydrolase
197	up	Cbei_2762	class IV aminotransferase
197	up	Cbei_2756	urea amidolyase-like protein
224	up	Cbei_1943	dihydropyrimidine dehydrogenase
233	up	Cbei_2760	hypothetical protein
235	up	Cbei_1999	nitrogenase iron protein
246	up	Cbei_2765	ABC transporter
275	up	Cbei_2768	inner-membrane translocator
325	up	Cbei_1940	binding-protein-dependent transport system innermembrane protein
368	up	Cbei_2769	extracellular ligand-binding receptor
4.5	down	Cbei_2659	pseudouridine synthase
4.5	down	Cbei_2610	3-methyl-2-oxobutanoatehydroxymethyltransferase
4.5	down	Cbei_0377	DNA-binding/iron metalloprotein/AP endonuclease
4.5	down	Cbei_0278	MarR family transcriptional regulator
4.5	down	Cbei_1636	hypothetical protein
4.5	down	Cbei_1405	hypothetical protein
4.5	down	Cbei_0195	ribonucleotide-diphosphate reductase subunitbeta
4.5	down	Cbei_0378	hypothetical protein
4.6	down	Cbei_3371	gluconate 5-dehydrogenase
4.6	down	Cbei_0419	ATP synthase F1 subunit epsilon
4.6	down	Cbei_2881	aldo/keto reductase
4.7	down	Cbei_0329	chaperonin GroEL
4.7	down	Cbei_5074	adenylosuccinate synthetase
4.7	down	Cbei_0200	hypothetical protein
4.7	down	Cbei_0285	glycogen/starch/alpha-glucan phosphorylase
4.7	down	Cbei_4440	hypothetical protein
4.8	down	Cbei_3105	dihydrouridine synthase
4.8	down	Cbei_3616	XRE family transcriptional regulator
4.8	down	Cbei_0201	hypothetical protein
4.9	down	Cbei_1311	NAD-binding 6-phosphogluconate dehydrogenase
4.9	down	Cbei_3912	VanZ family protein
4.9	down	Cbei_0657	type 11 methyltransferase
5	down	Cbei_4877	hypothetical protein
5	down	Cbei_3739	metallophosphoesterase
5	down	Cbei_3687	hypothetical protein

5.1	down	Cbei_2844	ABC transporter
5.1	down	Cbei_3565	two component transcriptional regulator
5.1	down	Cbei_3627	hypothetical protein
5.1	down	Cbei_0029	carbamoyl phosphate synthase large subunit
5.2	down	Cbei_1634	hypothetical protein
5.2	down	Cbei_3550	hypothetical protein
5.2	down	Cbei_3289	hypothetical protein
5.2	down	Cbei_0412	F0F1 ATP synthase subunit A
5.2	down	Cbei_1127	phosphate binding protein
5.2	down	Cbei_2951	two component transcriptional regulator
5.2	down	Cbei_1761	hypothetical protein
5.3	down	Cbei_0087	peptidyl-prolyl isomerase
5.3	down	Cbei_4704	glycoside hydrolase
5.3	down	Cbei_1646	hypothetical protein
5.4	down	Cbei_3688	hypothetical protein
5.4	down	Cbei_4593	hypothetical protein
5.5	down	Cbei_4771	hypothetical protein
5.5	down	Cbei_3628	DNA binding domain-containing protein
5.5	down	Cbei_1648	hypothetical protein
5.5	down	Cbei_3365	cell cycle protein
5.5	down	Cbei_1819	membrane-spanning protein
5.6	down	Cbei_2484	two component transcriptional regulator
5.7	down	Cbei_2997	hypothetical protein
5.7	down	Cbei_0416	F0F1 ATP synthase subunit alpha
5.7	down	Cbei_4842	xylose isomerase domain-containing protein
5.7	down	Cbei_1430	hypothetical protein
5.7	down	Cbei_2049	ABC transporter
5.7	down	Cbei_3567	UBA/Ts-N domain-containing protein
5.8	down	Cbei_4843	oxidoreductase domain-containing protein
5.9	down	Cbei_4714	hypothetical protein
6	down	Cbei_4124	methyl-accepting chemotaxis sensory transducer
6	down	Cbei_0346	nitroreductase
6	down	Cbei_0413	F0F1 ATP synthase subunit C
6.1	down	Cbei_1435	heavy metal transport/detoxification protein
6.2	down	Cbei_0281	hypothetical protein
6.2	down	Cbei_4619	oxidoreductase domain-containing protein
6.3	down	Cbei_2291	hypothetical protein
6.3	down	Cbei_2419	polyphosphate kinase
6.3	down	Cbei_2971	NLP/P60 protein
6.3	down	Cbei_0417	F0F1 ATP synthase subunit gamma
6.3	down	Cbei_0418	F0F1 ATP synthase subunit beta
6.3	down	Cbei_4523	nitroreductase
6.4	down	Cbei_3555	hypothetical protein
6.5	down	Cbei_0076	asparaginyl-tRNA synthetase
6.5	down	Cbei_2571	hypothetical protein
6.8	down	Cbei_3535	hypothetical protein
6.9	down	Cbei_3695	cobalt transport protein CbiM
6.9	down	Cbei_3549	XRE family transcriptional regulator

7	down	Cbei_0379	2-alkenal reductase
7	down	Cbei_4620	sugar transferase
7.1	down	Cbei_1516	ABC transporter
7.2	down	Cbei_4618	UTP-glucose-1-phosphate uridylyltransferase
7.2	down	Cbei_2482	hypothetical protein
7.2	down	Cbei_4439	thiamine biosynthesis protein ThiH
7.3	down	Cbei_3303	diguanylate cyclase/phosphodiesterase
7.3	down	Cbei_3557	TROVE domain-containing protein
7.4	down	Cbei_3106	heat shock protein DnaJ domain-containingprotein
7.4	down	Cbei_3556	hypothetical protein
7.4	down	Cbei_2670	hypothetical protein
7.5	down	Cbei_3623	hypothetical protein
7.5	down	Cbei_3575	hypothetical protein
7.6	down	Cbei_1494	hypothetical protein
7.7	down	Cbei_2135	peroxiredoxin
7.8	down	Cbei_4090	N-acetyltransferase GCN5
7.8	down	Cbei_2026	hypothetical protein
7.9	down	Cbei_1460	hypothetical protein
7.9	down	Cbei_2433	hypothetical protein
7.9	down	Cbei_3323	antibiotic transport-associated permeaseSpaG/MutG
8	down	Cbei_4717	cell wall binding repeat-containing protein
8.3	down	Cbei_2998	hypothetical protein
8.5	down	Cbei_0331	inosine 5'-monophosphate dehydrogenase
8.5	down	Cbei_2320	PTS system trehalose-specific transportersubunit IIBC
8.5	down	Cbei_2701	cell wall binding repeat-containing protein
8.7	down	Cbei_1729	glycosyl transferase family protein
8.7	down	Cbei_3286	hypothetical protein
8.9	down	Cbei_0328	co-chaperonin GroES
9.2	down	Cbei_3693	cobalt ABC transporter ATPase
9.2	down	Cbei_3354	NADPH-dependent FMN reductase
9.3	down	Cbei_2882	hypothetical protein
9.9	down	Cbei_3568	hypothetical protein
10	down	Cbei_2290	hypothetical protein
10	down	Cbei_2967	hypothetical protein
10	down	Cbei_0028	carbamoyl phosphate synthase small subunit
11	down	Cbei_2446	ornithine carbamoyltransferase
11	down	Cbei_3694	cobalt ABC transporter permease
11	down	Cbei_4669	glycoside hydrolase
11	down	Cbei_2483	hypothetical protein
12	down	Cbei_2946	hypothetical protein
12	down	Cbei_3649	MarR family transcriptional regulator
13	down	Cbei_3973	MerR family transcriptional regulator
13	down	Cbei_4113	alanine racemase
13	down	Cbei_4521	ornithine carbamoyltransferase
13	down	Cbei_1809	hypothetical protein
13	down	Cbei_2873	polysaccharide deacetylase
14	down	Cbei_4103	two component transcriptional regulator
14	down	Cbei_3569	RNA polymerase factor sigma-70

15	down	Cbei_3576	RNA polymerase factor sigma-70
15	down	Cbei_3536	ArsR family transcriptional regulator
16	down	Cbei_1425	permease
17	down	Cbei_4515	argininosuccinate synthase
18	down	Cbei_3763	periplasmic binding protein
18	down	Cbei_2981	hypothetical protein
18	down	Cbei_1820	hypothetical protein
18	down	Cbei_4517	N-acetyl-gamma-glutamyl-phosphate reductase
18	down	Cbei_4520	acetylornithine and succinylornithineaminotransferase
19	down	Cbei_3975	cell wall binding repeat-containing protein
19	down	Cbei_3974	aldo/keto reductase
19	down	Cbei_4519	acetylglutamate kinase
20	down	Cbei_1309	sodium:dicarboxylate symporter
20	down	Cbei_4623	cell wall binding repeat-containing protein
22	down	Cbei_1728	hypothetical protein
24	down	Cbei_4516	argininosuccinate lyase
25	down	Cbei_4518	bifunctional ornithineacetyltransferase/N-acetylglutamate synthase
26	down	Cbei_1806	hypothetical protein
29	down	Cbei_1808	ABC transporter
32	down	Cbei_1807	secretion protein HlyD family protein
33	down	Cbei_1308	"UDP-N-acetylmuramoylalanyl-D-glutamate--2,6-diaminopimelate ligase"
37	down	Cbei_3325	ABC transporter
50	down	Cbei_0538	hypothetical protein
57	down	Cbei_1436	peptidase S8/S53 subtilisin kexin sedolisin
61	down	Cbei_1725	glycosyl transferase family protein
67	down	Cbei_1726	hypothetical protein
77	down	Cbei_2445	hypothetical protein
84	down	Cbei_1138	hypothetical protein
98	down	Cbei_1724	glycosyl transferase family protein
117	down	Cbei_2188	hypothetical protein

Table S4B. Genes differentially expressed by more than 5 folds in DG-8052 in P2 medium with $CaCO_3$ vs DG-8052 in P2 medium during solventogensis.

Fold Change	up/ down	Gene Symbol	Gene Product
4.5	up	Cbei_0747	BNR repeat-containing glycosyl hydrolase
4.5	up	Cbei_4049	hypothetical protein
4.5	up	Cbei_2864	RND family efflux transporter MFP subunit
4.5	up	Cbei_1135	NAD(P)H-dependent glycerol-3-phosphatedehydrogenase
4.5	up	Cbei_0460	2-deoxy-D-gluconate 3-dehydrogenase
4.5	up	Cbei_1462	hypothetical protein
4.5	up	Cbei_4009	hypothetical protein
4.5	up	Cbei_3180	short-chain dehydrogenase/reductase SDR
4.5	up	Cbei_4341	hypothetical protein
4.5	up	Cbei_3825	ABC transporter permease
4.5	up	Cbei_3597	flavin reductase domain-containing protein
4.5	up	Cbei_3103	"peptidase M56, BlaR1"
4.5	up	Cbei_1502	carboxymuconolactone decarboxylase
4.6	up	Cbei_4127	dihydroxy-acid dehydratase
4.6	up	Cbei_0707	major facilitator superfamily transporter
4.6	up	Cbei_0238	hypothetical protein
4.6	up	Cbei_0296	hypothetical protein
4.6	up	Cbei_0391	peptidoglycan-binding LysM
4.7	up	Cbei_3027	PadR-like family transcriptional regulator
4.7	up	Cbei_1949	hypothetical protein
4.7	up	Cbei_2040	coenzyme A transferase
4.7	up	Cbei_2880	NAD-dependent epimerase/dehydratase
4.7	up	Cbei_4179	auxin efflux carrier
4.7	up	Cbei_1762	extracellular ligand-binding receptor
4.7	up	Cbei_0555	signal transduction histidine kinase LytS
4.7	up	Cbei_2409	hypothetical protein
4.7	up	Cbei_4190	sulfate ABC transporter ATPase
4.7	up	Cbei_4012	methyl-accepting chemotaxis sensory transducer
4.7	up	Cbei_3818	sensory histidine kinase AtoS
4.8	up	Cbei_4573	chorismate mutase
4.8	up	Cbei_2925	DEAD/DEAH box helicase
4.8	up	Cbei_2737	leucyl aminopeptidase
4.8	up	Cbei_2193	D-isomer specific 2-hydroxyacid dehydrogenaseNAD-binding subunit
4.8	up	Cbei_5050	membrane-bound proton-translocatingpyrophosphatase
4.8	up	Cbei_2518	aldehyde dehydrogenase
4.8	up	Cbei_0214	3-isopropylmalate dehydratase large subunit
4.9	up	Cbei_2093	multidrug ABC transporter ATPase
4.9	up	Cbei_0447	5-keto-4-deoxyuronate isomerase
4.9	up	Cbei_1766	ABC transporter
4.9	up	Cbei_2494	D-galactose-binding protein
4.9	up	Cbei_3182	alpha/beta fold family hydrolase

4.9	up	Cbei_3543	O-acetylhomoserineaminocarboxypropyltransferase
5	up	Cbei_2378	signal transduction histidine kinase LytS
5	up	Cbei_4217	type II secretion system protein
5	up	Cbei_0830	molecular chaperone DnaK
5	up	Cbei_0258	---
5	up	Cbei_4527	amino acid permease-associated protein
5	up	Cbei_3880	binding-protein-dependent transport system innermembrane protein
5	up	Cbei_0705	pyruvate formate-lyase
5.1	up	Cbei_3574	hypothetical protein
5.1	up	Cbei_3544	4Fe-4S ferredoxin
5.1	up	Cbei_5024	alpha/beta fold family hydrolase
5.1	up	Cbei_4468	GntR family transcriptional regulator
5.1	up	Cbei_2203	hypothetical protein
5.1	up	Cbei_3892	hypothetical protein
5.1	up	Cbei_2077	amidase
5.1	up	Cbei_1109	beta-lactamase domain-containing protein
5.1	up	Cbei_3451	transposase-like protein (20)
5.2	up	Cbei_3287	---
5.2	up	Cbei_1045	hypothetical protein
5.2	up	Cbei_0231	binding-protein-dependent transport system innermembrane protein
5.2	up	Cbei_3630	acetyl-CoA acetyltransferase
5.2	up	Cbei_2361	ArsR family transcriptional regulator
5.2	up	Cbei_3587	4Fe-4S ferredoxin
5.3	up	Cbei_2075	NUDIX hydrolase
5.3	up	Cbei_4082	glucuronate isomerase
5.3	up	Cbei_4641	sigma-54 factor interaction domain-containingprotein
5.3	up	Cbei_2196	PTS system mannose/fructose/sorbose familytransporter subunit IID
5.3	up	Cbei_4550	ribokinase-like domain-containing protein
5.3	up	Cbei_1483	hypothetical protein
5.3	up	Cbei_3133	LysR family transcriptional regulator
5.3	up	Cbei_4549	myo-inositol catabolism IolB domain-containingprotein
5.4	up	Cbei_0390	hypothetical protein
5.4	up	Cbei_0448	2-deoxy-D-gluconate 3-dehydrogenase
5.4	up	Cbei_4455	L-ribulose-5-phosphate 4-epimerase
5.4	up	Cbei_2390	GntR family transcriptional regulator
5.4	up	Cbei_2840	extradiol ring-cleavage dioxygenase III subunitB
5.4	up	Cbei_4679	binding-protein-dependent transport system innermembrane protein
5.4	up	Cbei_3459	4Fe-4S ferredoxin
5.5	up	Cbei_4083	mannitol dehydrogenase domain-containingprotein
5.5	up	Cbei_2571	hypothetical protein
5.5	up	Cbei_2852	isochorismatase hydrolase

5.5	up	Cbei_2853	major facilitator superfamily transporter
5.5	up	Cbei_2569	DNA mismatch repair protein MutS
5.5	up	Cbei_2345	"small acid-soluble spore protein, alpha/betatype"
5.6	up	Cbei_2094	multidrug ABC transporter
5.6	up	Cbei_2777	beta-lactamase domain-containing protein
5.6	up	Cbei_4923	MarR family transcriptional regulator
5.6	up	Cbei_2197	PTS system sorbose subfamily transporter subunitIIB
5.6	up	Cbei_1919	hypothetical protein
5.6	up	Cbei_3222	hypothetical protein
5.6	up	Cbei_1763	inner-membrane translocator
5.7	up	Cbei_4681	L-fucose isomerase-like protein
5.7	up	Cbei_4451	hypothetical protein
5.7	up	Cbei_2528	hypothetical protein
5.7	up	Cbei_4191	sulfate ABC transporter permease
5.8	up	Cbei_1918	"phosphotransferase system, EIIC"
5.8	up	Cbei_0452	L-rhamnose 1-epimerase
5.8	up	Cbei_1764	inner-membrane translocator
5.8	up	Cbei_4183	chemotaxis protein CheA
5.8	up	Cbei_1447	"small acid-soluble spore protein, alpha/betatype"
5.8	up	Cbei_3264	"small acid-soluble spore protein, alpha/betatype"
5.8	up	Cbei_3920	galactoside ABC transporter
5.9	up	Cbei_1593	OmpA/MotB domain-containing protein
5.9	up	Cbei_3562	transposase IS116/IS110/IS902 family protein
5.9	up	Cbei_1765	ABC transporter
5.9	up	Cbei_2348	hypothetical protein
5.9	up	Cbei_3223	hypothetical protein
6	up	Cbei_2389	L-fucose isomerase-like protein
6	up	Cbei_2838	MarR family transcriptional regulator
6	up	Cbei_0971	CopY family transcriptional regulator
6	up	Cbei_3483	XRE family transcriptional regulator
6	up	Cbei_3069	rubrerythrin
6.1	up	Cbei_1361	hypothetical protein
6.1	up	Cbei_4361	radical SAM domain-containing protein
6.1	up	Cbei_2738	transcriptional antiterminator BglG
6.2	up	Cbei_3028	major facilitator superfamily transporter
6.2	up	Cbei_2440	hypothetical protein
6.2	up	Cbei_2439	cytochrome b5
6.2	up	Cbei_1853	pyruvate flavodoxin/ferredoxin oxidoreductasedomain-containing protein
6.2	up	Cbei_2078	nicotinate phosphoribosyltransferase
6.3	up	Cbei_4193	sulfate ABC transporter substrate-bindingprotein
6.3	up	Cbei_1081	ABC transporter
6.3	up	Cbei_0748	deoxyribose-phosphate aldolase
6.3	up	Cbei_2110	arsenite-activated ATPase ArsA
6.3	up	Cbei_2214	---
6.3	up	Cbei_2321	"alpha,alpha-phosphotrehalase"
6.3	up	Cbei_3659	NAD-dependent epimerase/dehydratase
6.3	up	Cbei_2458	5-keto-4-deoxyuronate isomerase

6.4	up	Cbei_2377	periplasmic binding protein/LacI transcriptionalregulator
6.4	up	Cbei_2092	transcriptional regulator TrmB
6.4	up	Cbei_3448	hypothetical protein
6.4	up	Cbei_1254	ATP-dependent protease La
6.4	up	Cbei_3810	ROK family protein
6.5	up	Cbei_2199	PTS system fructose subfamily transportersubunit IIA
6.5	up	Cbei_5025	integral membrane sensor signal transductionhistidine kinase
6.6	up	Cbei_1128	phosphate ABC transporter permease
6.6	up	Cbei_4930	peptide ABC transporter ATPase
6.6	up	Cbei_2921	EmrB/QacA family drug resistance transporter
6.7	up	Cbei_3600	4Fe-4S ferredoxin
6.7	up	Cbei_3478	hypothetical protein
6.7	up	Cbei_2076	ribose-phosphate pyrophosphokinase
6.7	up	Cbei_5023	hypothetical protein
6.7	up	Cbei_1767	extracellular ligand-binding receptor
6.8	up	Cbei_3368	cupin
6.8	up	Cbei_1360	hypothetical protein
6.9	up	Cbei_2386	transaldolase
6.9	up	Cbei_1047	hypothetical protein
6.9	up	Cbei_2680	redoxin domain-containing protein
6.9	up	Cbei_0389	glutathione peroxidase
7	up	Cbei_2200	mannonate dehydratase
7	up	Cbei_2706	peptidase M24
7	up	Cbei_2079	---
7	up	Cbei_2115	DNA mismatch repair protein MutSdomain-containing protein
7	up	Cbei_1749	anthranilate synthase component I
7.1	up	Cbei_4016	histidine kinase
7.1	up	Cbei_2568	pentapeptide repeat-containing protein
7.2	up	Cbei_2111	protein tyrosine phosphatase
7.2	up	Cbei_4674	glycoside hydrolase family protein
7.2	up	Cbei_0257	malonyl CoA-acyl carrier protein transacylase
7.2	up	Cbei_1755	tryptophan synthase subunit alpha
7.4	up	Cbei_4505	cell wall binding repeat-containing protein
7.4	up	Cbei_1065	hypothetical protein
7.4	up	Cbei_2001	nitrogen regulatory protein P-II
7.4	up	Cbei_2932	pH-dependent sodium/proton antiporter
7.4	up	Cbei_0215	3-isopropylmalate dehydratase small subunit
7.7	up	Cbei_0749	RpiR family transcriptional regulator
7.7	up	Cbei_3629	hypothetical protein
7.7	up	Cbei_2061	ammonium transporter
7.8	up	Cbei_3893	MarR family transcriptional regulator
7.8	up	Cbei_3895	NADPH-dependent FMN reductase
7.8	up	Cbei_2198	hypothetical protein
7.9	up	Cbei_2081	hypothetical protein
7.9	up	Cbei_3482	peptide deformylase

7.9	up	Cbei_1011	formate acetyltransferase
8	up	Cbei_3153	hypothetical protein
8	up	Cbei_1441	binding-protein-dependent transport system innermembrane protein
8	up	Cbei_0110	"Mg2+ transporter protein, CorA family protein"
8.1	up	Cbei_2376	isochorismatase hydrolase
8.1	up	Cbei_4018	CheW protein
8.2	up	Cbei_1375	Ig domain-containing protein
8.2	up	Cbei_0462	2-dehydro-3-deoxyphosphogluconate aldolase
8.2	up	Cbei_0659	signal transduction histidine kinase regulatingcitrate/malate metabolism
8.2	up	Cbei_3814	beta-glucosidase
8.2	up	Cbei_0722	LysR family transcriptional regulator
8.2	up	Cbei_1046	hypothetical protein
8.3	up	Cbei_4454	transaldolase
8.3	up	Cbei_0652	tryptophan transport protein
8.3	up	Cbei_4645	transaldolase
8.5	up	Cbei_2524	cof family hydrolase
8.6	up	Cbei_3367	"sugar-specific permease, SgaT/UlaA"
8.6	up	Cbei_2201	2-dehydro-3-deoxyphosphogluconate aldolase
8.7	up	Cbei_3095	helix-turn-helix domain-containing protein
8.7	up	Cbei_2379	response regulator receiver protein
8.7	up	Cbei_4126	alpha-N-arabinofuranosidase
8.7	up	Cbei_4194	sulfate ABC transporter substrate-bindingprotein
8.8	up	Cbei_3184	RNA-directed DNA polymerase
8.8	up	Cbei_1129	phosphate ABC transporter permease
8.9	up	Cbei_2082	adenylosuccinate synthase
9	up	Cbei_0794	nicotinate-nucleotide pyrophosphorylase
9	up	Cbei_1255	hypothetical protein
9.1	up	Cbei_4508	FAD dependent oxidoreductase
9.1	up	Cbei_0739	NADPH-dependent FMN reductase
9.1	up	Cbei_2923	secretion protein HlyD family protein
9.1	up	Cbei_1751	anthranilate phosphoribosyltransferase
9.1	up	Cbei_5033	nitrogen regulatory protein P-II
9.2	up	Cbei_0461	ribokinase-like domain-containing protein
9.2	up	Cbei_3812	"phosphotransferase system,lactose/cellobiose-specific IIB subunit"
9.3	up	Cbei_1750	glutamine amidotransferase of anthranilatesynthase
9.3	up	Cbei_2113	hypothetical protein
9.3	up	Cbei_0703	hypothetical protein
9.4	up	Cbei_1752	Indole-3-glycerol phosphate synthase
9.4	up	Cbei_2213	"peptidase M48, Ste24p"
9.4	up	Cbei_5032	metal dependent phosphohydrolase
9.4	up	Cbei_3477	hypothetical protein
9.4	up	Cbei_3476	---
9.4	up	Cbei_3811	PTS system lactose/cellobiose-specifictransporter subunit IIA
9.5	up	Cbei_2225	"small acid-soluble spore protein, alpha/betatype"

9.5	up	Cbei_2041	citrate transporter
9.5	up	Cbei_4347	4-aminobutyrate aminotransferase
9.6	up	Cbei_2080	hypothetical protein
9.8	up	Cbei_4019	chemotaxis protein CheA
9.8	up	Cbei_0805	phage infection protein
9.9	up	Cbei_1459	methyl-accepting chemotaxis sensory transducer
10	up	Cbei_3735	XRE family transcriptional regulator
10	up	Cbei_1373	phage integrase family protein
10	up	Cbei_1446	pH-dependent sodium/proton antiporter
10	up	Cbei_2263	---
11	up	Cbei_4924	major facilitator superfamily transporter
11	up	Cbei_3813	PTS system lactose/cellobiose family transportersubunit IIC
11	up	Cbei_4450	monosaccharide-transporting ATPase
11	up	Cbei_4469	SCP-like extracellular protein
11	up	Cbei_4453	transketolase
11	up	Cbei_1754	tryptophan synthase subunit beta
11	up	Cbei_4644	transketolase
11	up	Cbei_2387	transketolase
11	up	Cbei_3277	class II aldolase/adducin family protein
11	up	Cbei_0532	transketolase
11	up	Cbei_3733	hypothetical protein
11	up	Cbei_2357	extracellular solute-binding protein
11	up	Cbei_4799	sugar isomerase (SIS)
11	up	Cbei_2000	nitrogen regulatory protein P-II
11	up	Cbei_1442	binding-protein-dependent transport system innermembrane protein
11	up	Cbei_3032	hypothetical protein
12	up	Cbei_1937	iron-containing alcohol dehydrogenase
12	up	Cbei_2011	isopropylmalate/homocitrate/citramalatesynthase-like protein
12	up	Cbei_0653	polysaccharide deacetylase
12	up	Cbei_0218	ketol-acid reductoisomerase
12	up	Cbei_2870	glycine/betaine ABC transportersubstrate-binding protein
12	up	Cbei_4929	peptide ABC transporter ATPase
13	up	Cbei_4080	histidine triad (HIT) protein
13	up	Cbei_4675	glycoside hydrolase
13	up	Cbei_1351	hypothetical protein
13	up	Cbei_3278	coenzyme A transferase
13	up	Cbei_0777	glutathione peroxidase
13	up	Cbei_3730	hypothetical protein
13	up	Cbei_4676	response regulator receiver protein
13	up	Cbei_3038	"peptidase, membrane zinc metallopeptidase"
14	up	Cbei_0262	ABC transporter
14	up	Cbei_4507	FAD-dependent pyridine nucleotide-disulfideoxidoreductase
14	up	Cbei_4548	"thiamine pyrophosphate protein, central region"
14	up	Cbei_3036	cell wall binding repeat-containing protein

14	up	Cbei_2839	methyl-accepting chemotaxis sensory transducer
14	up	Cbei_4457	L-arabinose isomerase
14	up	Cbei_4350	extracellular solute-binding protein
14	up	Cbei_3096	ABC transporter
14	up	Cbei_0721	phage replisome organizer
14	up	Cbei_3734	hypothetical protein
14	up	Cbei_0216	3-isopropylmalate dehydrogenase
15	up	Cbei_2754	"acetyl-CoA carboxylase, biotin carboxylase"
15	up	Cbei_2083	GntR family transcriptional regulator
15	up	Cbei_1063	"peptidase M56, BlaR1"
15	up	Cbei_1130	phosphate ABC transporter ATPase
15	up	Cbei_4772	methyl-accepting chemotaxis sensory transducer
15	up	Cbei_1753	phosphoribosylanthranilate isomerase
15	up	Cbei_0451	oligogalacturonide transporter
15	up	Cbei_4942	nitrogen regulatory protein P-II
16	up	Cbei_4173	ABC transporter
16	up	Cbei_2871	glycine/betaine ABC transporter ATPase
16	up	Cbei_3737	NADPH-dependent FMN reductase
16	up	Cbei_0738	major facilitator superfamily transporter
16	up	Cbei_4547	oxidoreductase domain-containing protein
16	up	Cbei_2112	arsenical-resistance protein
16	up	Cbei_4352	nitrilase/cyanide hydratase and apolipoproteinN-acyltransferase
16	up	Cbei_4934	putrescine--2-oxoglutarate aminotransferase
16	up	Cbei_1444	alpha amylase
16	up	Cbei_2012	homocitrate synthase
17	up	Cbei_4506	hypothetical protein
17	up	Cbei_3037	ATP-dependent metalloprotease FtsH
17	up	Cbei_1131	phosphate uptake regulator PhoU
17	up	Cbei_4081	oligogalacturonide transporter
17	up	Cbei_0444	glutamine synthetase
17	up	Cbei_2358	binding-protein-dependent transport system innermembrane protein
17	up	Cbei_1443	Alpha-glucosidase
18	up	Cbei_4928	extracellular solute-binding protein
18	up	Cbei_2922	secretion protein HlyD
18	up	Cbei_0217	acetolactate synthase large subunit
19	up	Cbei_0260	EmrB/QacA family drug resistance transporter
19	up	Cbei_3521	hypothetical protein
20	up	Cbei_1132	phosphate uptake regulator PhoU
20	up	Cbei_4452	carbohydrate kinase FGGY
21	up	Cbei_2570	hypothetical protein
21	up	Cbei_2459	2-keto-3-deoxygluconate permease
21	up	Cbei_1833	altronate oxidoreductase
21	up	Cbei_4458	histidine kinase internal region
22	up	Cbei_3517	ROK family protein
23	up	Cbei_4546	xylose isomerase domain-containing protein
23	up	Cbei_4542	acyl-CoA dehydrogenase domain-containingprotein

23	up	Cbei_1944	allantoate amidohydrolase
23	up	Cbei_2344	hypothetical protein
23	up	Cbei_1998	hypothetical protein
24	up	Cbei_0449	glycosyl hydrolase family protein
25	up	Cbei_2758	alanine racemase
25	up	Cbei_4351	ABC transporter
26	up	Cbei_0111	amidohydrolase
26	up	Cbei_2359	monosaccharide-transporting ATPase
26	up	Cbei_2384	xylulokinase
27	up	Cbei_4172	extracellular solute-binding protein
27	up	Cbei_3651	major facilitator superfamily transporter
27	up	Cbei_2391	extracellular solute-binding protein
27	up	Cbei_2383	xylose isomerase
28	up	Cbei_1999	nitrogenase iron protein
29	up	Cbei_1997	hypothetical protein
30	up	Cbei_1832	glucuronate isomerase
30	up	Cbei_1943	dihydropyrimidine dehydrogenase
31	up	Cbei_3731	hypothetical protein
31	up	Cbei_0264	hypothetical protein
31	up	Cbei_1941	NMT1/THI5-like protein
31	up	Cbei_2006	"ferredoxin, 2Fe-2S"
32	up	Cbei_4545	sugar transporter
32	up	Cbei_2388	hypothetical protein
33	up	Cbei_3876	ribokinase-like domain-containing protein
33	up	Cbei_2003	nitrogenase molybdenum-iron protein subunitbeta
33	up	Cbei_3185	methionine--tRNA ligase
34	up	Cbei_5034	ammonium transporter
34	up	Cbei_3736	major facilitator superfamily transporter
35	up	Cbei_0109	sugar transporter
35	up	Cbei_0317	transaldolase
35	up	Cbei_0263	hypothetical protein
35	up	Cbei_4171	polar amino acid ABC transporter inner membranesubunit
36	up	Cbei_2360	alpha-N-arabinofuranosidase
37	up	Cbei_3518	aldose 1-epimerase
38	up	Cbei_0261	RND family efflux transporter MFP subunit
38	up	Cbei_4543	coenzyme A transferase
38	up	Cbei_4170	polar amino acid ABC transporter inner membranesubunit
39	up	Cbei_4449	ABC transporter
40	up	Cbei_4936	aldehyde dehydrogenase
40	up	Cbei_0315	pyruvate formate-lyase
41	up	Cbei_3519	hydantoinase/oxoprolinase
41	up	Cbei_1458	pyruvate flavodoxin/ferredoxin oxidoreductasedomain-containing protein
42	up	Cbei_3522	citrate transporter
44	up	Cbei_0265	hypothetical protein
45	up	Cbei_3523	hypothetical protein

51	up	Cbei_4940	spermidine/putrescine ABC transporter ATPase
53	up	Cbei_4125	sugar (glycoside-Pentoside-hexuronide)transporter
53	up	Cbei_0266	hypothetical protein
54	up	Cbei_3878	extracellular solute-binding protein
55	up	Cbei_1834	histidine triad (HIT) protein
60	up	Cbei_0701	glycyl-radical activating family protein
61	up	Cbei_0316	glycyl-radical activating family protein
73	up	Cbei_4448	monosaccharide-transporting ATPase
76	up	Cbei_1598	RpiR family transcriptional regulator
81	up	Cbei_2343	AsnC family transcriptional regulator
81	up	Cbei_5059	methyl-accepting chemotaxis sensory transducer
83	up	Cbei_0306	ATPase P
84	up	Cbei_1838	altronate oxidoreductase
87	up	Cbei_0454	rhamnulose-1-phosphate aldolase
93	up	Cbei_1836	keto-hydroxyglutarate-aldolase/keto-deoxy-phosp hogluconate aldolase
94	up	Cbei_2757	allophanate hydrolase subunit 1
94	up	Cbei_0455	"L-1,2-propanediol oxidoreductase"
105	up	Cbei_0318	mannose-1-phosphate guanylyltransferase (GDP)
105	up	Cbei_1837	ribokinase-like domain-containing protein
107	up	Cbei_2755	biotin/lipoyl attachment domain-containingprotein
116	up	Cbei_2759	LamB/YcsF family protein
118	up	Cbei_1839	altronate dehydratase
137	up	Cbei_2761	hypothetical protein
164	up	Cbei_1835	sugar (glycoside-Pentoside-hexuronide)transporter
189	up	Cbei_2760	hypothetical protein
243	up	Cbei_2342	aspartate ammonia-lyase
246	up	Cbei_2762	class IV aminotransferase
4.5	down	Cbei_1539	adenine phosphoribosyltransferase
4.5	down	Cbei_1032	RNA-binding S1 domain-containing protein
4.5	down	Cbei_1445	hypothetical protein
4.5	down	Cbei_3656	iron-sulfur flavoprotein
4.5	down	Cbei_2576	cof family hydrolase
4.6	down	Cbei_3075	PAS/PAC sensor-containing diguanylatecyclase/phosphodiesterase
4.6	down	Cbei_1088	"membrane bound O-acyl transferase, MBOAT familyprotein"
4.6	down	Cbei_4925	PAS/PAC sensor signal transduction histidinekinase
4.6	down	Cbei_3138	cupin
4.6	down	Cbei_1224	riboflavin biosynthesis protein RibD
4.6	down	Cbei_1153	ribulose-phosphate 3-epimerase
4.6	down	Cbei_1344	hypothetical protein
4.6	down	Cbei_1078	HutP protein
4.6	down	Cbei_R0070	5S ribosomal RNA

4.6	down	Cbei_0406	Sua5/YciO/YrdC/YwlC family protein
4.7	down	Cbei_0599	triosephosphate isomerase
4.7	down	Cbei_1315	histidine--tRNA ligase
4.7	down	Cbei_5100	single-stranded nucleic acid binding R3Hdomain-containing protein
4.7	down	Cbei_1146	methionyl-tRNA formyltransferase
4.7	down	Cbei_3323	antibiotic transport-associated permeaseSpaG/MutG
4.7	down	Cbei_2029	K+ potassium transporter
4.7	down	Cbei_0996	mannose-6-phosphate isomerase
4.7	down	Cbei_5028	1-acyl-sn-glycerol-3-phosphate acyltransferase
4.7	down	Cbei_2232	serine dehydratase subunit alpha
4.7	down	Cbei_1911	hypothetical protein
4.7	down	Cbei_2887	"electron transfer flavoprotein,alpha/beta-subunit-like protein"
4.8	down	Cbei_0598	phosphoglycerate kinase
4.8	down	Cbei_1151	protein kinase
4.8	down	Cbei_0439	RNA-binding S1 domain-containing protein
4.8	down	Cbei_4851	pyruvate kinase
4.8	down	Cbei_0499	cell cycle protein
4.8	down	Cbei_3105	dihydrouridine synthase
4.8	down	Cbei_2179	hypothetical protein
4.8	down	Cbei_3063	hypothetical protein
4.8	down	Cbei_2883	acyl-CoA dehydrogenase domain-containingprotein
4.8	down	Cbei_2484	two component transcriptional regulator
4.9	down	Cbei_4725	cell wall binding repeat-containing protein
4.9	down	Cbei_0509	hypothetical protein
4.9	down	Cbei_2845	two component transcriptional regulator
4.9	down	Cbei_2884	FAD linked oxidase domain-containing protein
4.9	down	Cbei_3398	phage-like element pbsx protein XkdK
4.9	down	Cbei_1799	type 11 methyltransferase
4.9	down	Cbei_4289	flagellin domain-containing protein
4.9	down	Cbei_3197	glycosyl transferase family protein
4.9	down	Cbei_0407	RpiB/LacA/LacB family sugar-phosphate isomerase
5	down	Cbei_R0030	tRNA-Cys
5	down	Cbei_2182	ferredoxin-NADP(+) reductase subunit alpha
5	down	Cbei_4394	hypothetical protein
5	down	Cbei_0312	FAD linked oxidase domain-containing protein
5.1	down	Cbei_3397	phage-like element pbsx protein XkdM
5.1	down	Cbei_3219	sodium:dicarboxylate symporter
5.1	down	Cbei_1575	cell division protein MraZ
5.1	down	Cbei_3964	CHAP domain-containing protein
5.1	down	Cbei_1391	methyl-accepting chemotaxis sensory transducer
5.1	down	Cbei_3076	diguanylate cyclase
5.1	down	Cbei_0368	polysaccharide deacetylase
5.1	down	Cbei_0489	Maf-like protein
5.2	down	Cbei_2424	diguanylate cyclase/phosphodiesterase

5.2	down	Cbei_4391	hypothetical protein
5.2	down	Cbei_2967	hypothetical protein
5.2	down	Cbei_4665	glycoside hydrolase
5.2	down	Cbei_0597	"glyceraldehyde-3-phosphate dehydrogenase, typeI"
5.2	down	Cbei_2659	pseudouridine synthase
5.2	down	Cbei_0508	50S ribosomal protein L21
5.2	down	Cbei_2183	oxidoreductase
5.2	down	Cbei_2639	hypothetical protein
5.3	down	Cbei_1343	hypothetical protein
5.3	down	Cbei_0596	DeoR family transcriptional regulator
5.3	down	Cbei_0491	rod shape-determining protein MreB
5.3	down	Cbei_4832	methyl-accepting chemotaxis sensory transducer
5.4	down	Cbei_0418	F0F1 ATP synthase subunit beta
5.4	down	Cbei_1226	GTP cyclohydrolase II
5.4	down	Cbei_R0074	tRNA-Gln
5.4	down	Cbei_1724	glycosyl transferase family protein
5.5	down	Cbei_1775	phosphate transporter
5.5	down	Cbei_1400	glycosyl transferase family protein
5.5	down	Cbei_4392	hypothetical protein
5.5	down	Cbei_0196	phosphoenolpyruvate-protein phosphotransferase
5.5	down	Cbei_4403	hypothetical protein
5.6	down	Cbei_1425	permease
5.6	down	Cbei_0301	biotin synthase
5.6	down	Cbei_0118	4Fe-4S ferredoxin
5.6	down	Cbei_4670	6-phospho-beta-glucosidase
5.7	down	Cbei_3568	hypothetical protein
5.7	down	Cbei_4839	aminoglycoside N(3')-acetyltransferase
5.7	down	Cbei_0419	ATP synthase F1 subunit epsilon
5.7	down	Cbei_R0042	tRNA-Lys
5.7	down	Cbei_R0088	5S ribosomal RNA
5.7	down	Cbei_0356	deoxyribonucleotide triphosphatepyrophosphatase
5.7	down	Cbei_1819	membrane-spanning protein
5.8	down	Cbei_1637	hypothetical protein
5.8	down	Cbei_4709	UDP-3-O-(R-3-hydroxymyristoyl)-glucosamineN -acyltransferase
5.8	down	Cbei_1145	peptide deformylase
5.8	down	Cbei_4524	NADPH-dependent FMN reductase
5.8	down	Cbei_4325	glycosyl transferase family protein
5.9	down	Cbei_0028	carbamoyl phosphate synthase small subunit
5.9	down	Cbei_1225	riboflavin synthase subunit alpha
5.9	down	Cbei_1237	S-ribosylhomocysteinase
5.9	down	Cbei_1899	glyoxalase/bleomycin resistanceprotein/dioxygenase
6	down	Cbei_0357	phosphodiesterase
6	down	Cbei_0510	50S ribosomal protein L27
6	down	Cbei_1227	"6,7-dimethyl-8-ribityllumazine synthase"
6	down	Cbei_4718	cell wall binding repeat-containing protein

6.1	down	Cbei_3688	hypothetical protein
6.1	down	Cbei_1641	hypothetical protein
6.1	down	Cbei_2882	hypothetical protein
6.1	down	Cbei_3616	XRE family transcriptional regulator
6.1	down	Cbei_5003	cyclopropane-fatty-acyl-phospholipid synthase
6.1	down	Cbei_4407	hypothetical protein
6.1	down	Cbei_4714	hypothetical protein
6.2	down	Cbei_1640	hypothetical protein
6.2	down	Cbei_4841	oxidoreductase domain-containing protein
6.2	down	Cbei_4483	beta-lactamase domain-containing protein
6.2	down	Cbei_3693	cobalt ABC transporter ATPase
6.2	down	Cbei_5104	50S ribosomal protein L34
6.2	down	Cbei_1635	phage-related terminase small subunit-likeprotein
6.2	down	Cbei_2475	TetR family transcriptional regulator
6.3	down	Cbei_4629	polysaccharide biosynthesis protein CapD
6.3	down	Cbei_4523	nitroreductase
6.4	down	Cbei_2166	hypothetical protein
6.4	down	Cbei_0982	ribosomal 5S rRNA E-loop binding proteinCtc/L25/TL5
6.4	down	Cbei_1316	ATP phosphoribosyltransferase
6.4	down	Cbei_0995	hypothetical protein
6.4	down	Cbei_4852	6-phosphofructokinase
6.4	down	Cbei_3106	heat shock protein DnaJ domain-containingprotein
6.4	down	Cbei_1396	BAAT/Acyl-CoA thioester hydrolase-like protein
6.5	down	Cbei_4335	ABC transporter
6.6	down	Cbei_R0057	tRNA-Tyr
6.6	down	Cbei_1644	hypothetical protein
6.6	down	Cbei_4623	cell wall binding repeat-containing protein
6.8	down	Cbei_4090	N-acetyltransferase GCN5
6.8	down	Cbei_4910	N-acetyltransferase GCN5
7	down	Cbei_R0041	tRNA-Gln
7	down	Cbei_0029	carbamoyl phosphate synthase large subunit
7.1	down	Cbei_4840	helix-turn-helix domain-containing protein
7.1	down	Cbei_3694	cobalt ABC transporter permease
7.1	down	Cbei_1313	thiamine biosynthesis protein ThiI
7.1	down	Cbei_4487	thiamine-phosphate pyrophosphorylase
7.2	down	Cbei_1382	histidine kinase internal region
7.2	down	Cbei_1809	hypothetical protein
7.3	down	Cbei_4691	polysaccharide biosynthesis protein
7.3	down	Cbei_3569	RNA polymerase factor sigma-70
7.4	down	Cbei_0415	F0F1 ATP synthase subunit delta
7.5	down	Cbei_0401	50S ribosomal protein L31
7.5	down	Cbei_4619	oxidoreductase domain-containing protein
7.5	down	Cbei_2968	DoxX family protein
7.5	down	Cbei_0416	F0F1 ATP synthase subunit alpha
7.6	down	Cbei_3576	RNA polymerase factor sigma-70
7.9	down	Cbei_4760	cell wall binding repeat-containing protein
7.9	down	Cbei_4628	hypothetical protein

7.9	down	Cbei_4843	oxidoreductase domain-containing protein
7.9	down	Cbei_0414	F0F1 ATP synthase subunit B
7.9	down	Cbei_0516	RluA family pseudouridine synthase
8	down	Cbei_2330	"methionine synthase, vitamin-B12 independent"
8	down	Cbei_4490	phosphomethylpyrimidine kinase
8	down	Cbei_3973	MerR family transcriptional regulator
8.1	down	Cbei_1761	hypothetical protein
8.2	down	Cbei_3395	hypothetical protein
8.2	down	Cbei_4488	hydroxyethylthiazole kinase
8.3	down	Cbei_2670	hypothetical protein
8.3	down	Cbei_0417	F0F1 ATP synthase subunit gamma
8.3	down	Cbei_3623	hypothetical protein
8.5	down	Cbei_3687	hypothetical protein
8.5	down	Cbei_4489	thiamine biosynthesis protein ThiC
8.5	down	Cbei_0378	hypothetical protein
8.7	down	Cbei_1155	50S ribosomal protein L28
8.7	down	Cbei_1436	peptidase S8/S53 subtilisin kexin sedolisin
8.7	down	Cbei_2483	hypothetical protein
8.9	down	Cbei_1533	queuine tRNA-ribosyltransferase
8.9	down	Cbei_0377	DNA-binding/iron metalloprotein/AP endonuclease
9	down	Cbei_1642	hypothetical protein
9	down	Cbei_4618	UTP-glucose-1-phosphate uridylyltransferase
9	down	Cbei_4842	xylose isomerase domain-containing protein
9.1	down	Cbei_1149	ribosomal RNA large subunit methyltransferase N
9.3	down	Cbei_2545	4-hydroxy-3-methylbut-2-enyl diphosphatereductase
9.4	down	Cbei_3695	cobalt transport protein CbiM
9.5	down	Cbei_3171	accessory gene regulator B
9.6	down	Cbei_0346	nitroreductase
9.6	down	Cbei_3974	aldo/keto reductase
9.7	down	Cbei_4405	hypothetical protein
9.7	down	Cbei_4669	glycoside hydrolase
9.9	down	Cbei_0413	F0F1 ATP synthase subunit C
10	down	Cbei_0412	F0F1 ATP synthase subunit A
10	down	Cbei_1395	MarR family transcriptional regulator
10	down	Cbei_2028	two component transcriptional regulator
10	down	Cbei_2482	hypothetical protein
10	down	Cbei_1494	hypothetical protein
10	down	Cbei_1310	RelA/SpoT domain-containing protein
11	down	Cbei_1495	hypothetical protein
11	down	Cbei_0656	deoxyuridine 5'-triphosphate nucleotidohydrolaseDut
11	down	Cbei_1516	ABC transporter
11	down	Cbei_4406	hypothetical protein
11	down	Cbei_4620	sugar transferase
11	down	Cbei_4491	thiW protein
11	down	Cbei_3975	cell wall binding repeat-containing protein

11	down	Cbei_4521	ornithine carbamoyltransferase
11	down	Cbei_4689	hypothetical protein
11	down	Cbei_0486	aminoacyl-histidine dipeptidase
12	down	Cbei_3354	NADPH-dependent FMN reductase
12	down	Cbei_4408	carboxynorspermidine decarboxylase
13	down	Cbei_4690	membrane spanning protein
13	down	Cbei_0280	hypothetical protein
13	down	Cbei_3755	hypothetical protein
13	down	Cbei_4103	two component transcriptional regulator
13	down	Cbei_4113	alanine racemase
14	down	Cbei_4694	nucleotidyl transferase
14	down	Cbei_2445	hypothetical protein
14	down	Cbei_4339	nucleotidyl transferase
14	down	Cbei_2181	iron-containing alcohol dehydrogenase
15	down	Cbei_3912	VanZ family protein
15	down	Cbei_3324	lantibiotic permease spaE/mutE
15	down	Cbei_1649	Phage XkdN-like protein
16	down	Cbei_1807	secretion protein HlyD family protein
16	down	Cbei_3286	hypothetical protein
16	down	Cbei_4695	choline/ethanolamine kinase
16	down	Cbei_4520	acetylornithine and succinylornithineaminotransferase
16	down	Cbei_2981	hypothetical protein
17	down	Cbei_1722	iron-containing alcohol dehydrogenase
17	down	Cbei_4519	acetylglutamate kinase
17	down	Cbei_4517	N-acetyl-gamma-glutamyl-phosphate reductase
17	down	Cbei_1808	ABC transporter
18	down	Cbei_4336	class V aminotransferase
18	down	Cbei_3325	ABC transporter
18	down	Cbei_2027	osmosensitive K+ channel signal transductionhistidine kinase
19	down	Cbei_3396	Phage XkdN-like protein
19	down	Cbei_1806	hypothetical protein
19	down	Cbei_0538	hypothetical protein
19	down	Cbei_2842	hypothetical protein
20	down	Cbei_4518	bifunctional ornithineacetyltransferase/N-acetylglutamate synthase
21	down	Cbei_4337	thiamine pyrophosphate binding domain-containingprotein
22	down	Cbei_2843	hypothetical protein
23	down	Cbei_4516	argininosuccinate lyase
23	down	Cbei_1311	NAD-binding 6-phosphogluconate dehydrogenase
26	down	Cbei_4515	argininosuccinate synthase
33	down	Cbei_2982	hypothetical protein
34	down	Cbei_1138	hypothetical protein
35	down	Cbei_1820	hypothetical protein
35	down	Cbei_4717	cell wall binding repeat-containing protein

37	down	Cbei_4338	cytidyltransferase-like protein
43	down	Cbei_2844	ABC transporter
44	down	Cbei_1309	sodium:dicarboxylate symporter
57	down	Cbei_1308	"UDP-N-acetylmuramoylalanyl-D-glutamate--2,6 -diaminopimelate ligase"
61	down	Cbei_0657	type 11 methyltransferase
85	down	Cbei_2188	hypothetical protein

Table S5A. Fold change of solvent production genes according to microarray analysis

Gene Symbol	Protein/Enzyme	Fold Change	
		acidogenisis	solventogensis
Cbei_3516	pyruvate synthase	-2.70	-1.57
Cbei_2063		+1.15	+2.98
Cbei_1853	pyruvate/ferredoxin oxidoreductase	+3.58	+6.21
Cbei_1458		+2.01	+41.18
Cbei_4042		+2.60	+3.11
Cbei_1165	acetate kinase	-2.33	-1.19
Cbei_1164	phosphotransacetylase	-2.37	-1.67
Cbei_1953	aldehyde dehydrogenase	+2.43	-1.22
Cbei_3832		-1.37	-1.40
Cbei_2518		+4.33	+4.83
Cbei_0305		+3.36	+4.01
Cbei_4045		+1.5	+2.28
Cbei_4936		+164.7	+39.86
Cbei_0729		+1.21	+3.17
Cbei_1932	alcohol dehydrogenase	-1.98	-2.56
Cbei_4552		+3.42	+5.31
Cbei_0223		+1.21	-1.01
Cbei_0305		+3.36	+4.01
Cbei_4354		+8.44	+3.0
Cbei_2194		+1.35	+4.44
Cbei_1937		+159.77	+11.52
Cbei_0455		+1.95	+93.9
Cbei_0528		+2.73	+4.18
Cbei_3630	thiolase (acetyl-CoA acetyltransferase)	+2.12	+5.19
Cbei_3835	acetoacetate decarboxylase	+1.01	+1.10
Cbei_3819	acetoacetyl-CoA transferase subunit A	-1.56	-1.57
Cbei_2040		+3.40	+4.69
Cbei_3278		+3.99	+12.99
Cbei_3833	butyrate-acetoacetate CoA-transferase subunit A	-1.27	-1.0
Cbei_0325	3-hydroxybutyryl-CoA dehydrogenase	+1.26	-1.01
Cbei_2231	Enoyl-CoA hydratase	-1.06	-2.27
Cbei_2883	butyryl-CoA dehydrogenase	+3.17	-4.85
Cbei_3820		-1.12	+1.80
Cbei_4609	butyrate kinase	+1.01	-1.95
Cbei_4006		+6.05	-1.39
Cbei_4899	phosphotrans butyrylase	-2.5	-1.19

Table S5B. Fold change of sugar transporter genes according to microarray analysis

Cbei Symbol	Protein/Enzyme	Fold Change (Acidogenesis)	Fold Change (Solventogenesis)
two-component system			
Cbei_0444	glutamine synthetase	+9.45	+17
Cbei_0660	accessory gene regulator B	-2.59	-2.55
Cbei_1127	phosphate binding protein	-5.2	+4.38
Cbei_1749	anthranilate synthase component I	+8.11	+7.03
Cbei_1752	Indole-3-glycerol phosphate synthase	ND*	+9.36
Cbei_3630	acetyl-CoA acetyltransferase	+2.12	+5.19
Cbei_4008	malate dehydrogenase	+44	ND*
Cbei_4015	response regulator receiver protein	+2.34	+3.15
Cbei_4018	CheW protein	+3.42	+8.11
Cbei_4174	response regulator receiver	+2.03	ND*
Cbei_4180	response regulator receiver modulated CheB methylesterase	+6.24	+4.01
Cbei_4181	protein-glutamate O-methyltransferase	+3.33	+3.5
Cbei_4183	chemotaxis protein CheA	+5.3	+5.8
Cbei_4184	CheW protein	ND*	+2.86
Cbei_4206	glutamine synthetase, type I	+6.46	+2.93
Cbei_4308	protein-glutamate O-methyltransferase	+2.8	ND*
Cbei_4311	CheW protein	ND*	+2.51
Cbei_4430	multi-sensor signal transduction histidine kinase	+2.21	ND*
Cbei_4458	histidine kinase internal region	+2.05	+21.4
Cbei_4579	sensor histidine kinase-like protein	ND*	+2.85
Cbei_4942	nitrogen regulatory protein P-II	+130	+2.13
Cbei_5033	nitrogen regulatory protein P-II	+8.65	+9.13
ABC transporter			
Cbei_0638	YaeC family lipoprotein	+2.09	+3.7

Cbei_1762	extracellular ligand-binding receptor	+5.57	+4.7
Cbei_1763	inner-membrane translocator	+6.33	+5.63
Cbei_1764	inner-membrane translocator	+4.5	+5.8
Cbei_1765	ABC transporter	+3.97	+5.87
Cbei_1766	ABC transporter	+5.86	+4.88
Cbei_1767	extracellular ligand-binding receptor	+3.62	+6.72
Cbei_1938	ABC transporter	+166	ND*
Cbei_1940	binding-protein-dependent transport system innermembrane protein	+325	ND*
Cbei_1941	NMT1/THI5-like protein	+112	+30.9
Cbei_1992	molybdenum ABC transporter periplasmicmolybdate-binding protein	+8.27	ND*
Cbei_2394	ABC transporter	+2.53	+3.96
Cbei_2765	ABC transporter	+246	ND*
Cbei_2767	inner-membrane translocator	+161	ND*
Cbei_2768	inner-membrane translocator	+275	ND*
Cbei_2769	extracellular ligand-binding receptor	+368	ND*
Cbei_2870	glycine/betaine ABC transportersubstrate-binding protein	+2.98	+12.2
Cbei_3703	transport system permease	+4.93	+2.71
Cbei_3704	ABC transporter	+2.13	ND*
Cbei_4432	monosaccharide-transporting ATPase	+4.92	ND*
Cbei_4433	ABC transporter	+10.1	ND*
Cbei_4928	extracellular solute-binding protein	+12.3	+18.1
Cbei_4929	peptide ABC transporter ATPase	+15.8	+12.3
Cbei_4930	peptide ABC transporter ATPase	+12.1	+6.64
Cbei_4931	binding-protein-dependent transport system innermembrane protein	+11.8	+3.6
Cbei_4932	binding-protein-dependent transport system innermembrane protein	+9.66	+4.43
Cbei_4940	spermidine/putrescine ABC transporter ATPase	+147	+50.8
Cbei_4941	extracellular solute-binding protein	+158	+15.3
Cbei_5042	extracellular ligand-binding receptor	+11.5	ND*
Cbei_5043	inner-membrane translocator	+8.67	ND*

Puryvate metabolism			
Cbei_1076	acetyl-CoA carboxylase, carboxyl transferasesubunit beta	+2.27	ND*
Cbei_0213	alpha-isopropylmalate/homocitrate synthasefamily transferase	+19.9	+4.32
Cbei_0305	bifunctional acetaldehyde-CoA/alcoholdehydrogenase	+3.36	+4.01
Cbei_0315	pyruvate formate-lyase	ND*	+40.3
Cbei_0455	L-1,2-propanediol oxidoreductase	ND*	+93.9
Cbei_0705	pyruvate formate-lyase	ND*	+5.01
Cbei_1011	formate acetyltransferase	+7.41	+7.95
Cbei_2012	homocitrate synthase	+41.4	16.4
Cbei_2063	phosphoenolpyruvate synthase	ND*	+2.98
Cbei_2754	acetyl-CoA carboxylase, biotin carboxylase	+32	+14.6
Cbei_2789	L-lactate dehydrogenase	+7.02	+3.84
Cbei_3630	acetyl-CoA acetyltransferase	+2.12	+5.19
Cbei_4008	malate dehydrogenase	+44	ND*
Cbei_4045	aldehyde dehydrogenase	ND*	+2.28
Cbei_4903	L-lactate dehydrogenase	+3.01	-2.06
Cbei_4960	pyruvate carboxylase	ND*	+2.46
Pentose and glucuronate interconversions			
Cbei_0447	5-keto-4-deoxyuronate isomerase	+2.01	+4.87
Cbei_0454	rhamnulose-1-phosphate aldolase	ND*	+87.5
Cbei_0460	2-deoxy-D-gluconate 3-dehydrogenase	ND*	+4.49
Cbei_0461	ribokinase-like domain-containing protein	ND*	+9.71
Cbei_0462	2-dehydro-3-deoxyphosphogluconate aldolase	ND*	+8.15
Cbei_1832	glucuronate isomerase	ND*	+29.9
Cbei_1833	altronate oxidoreductase	ND*	+21.3
Cbei_1836	keto-hydroxyglutarate-aldolase/keto-deoxy-phosphogluconate aldolase	ND*	+92.5
Cbei_1837	ribokinase-like domain-containing protein	ND*	+105
Cbei_1838	altronate oxidoreductase	ND*	+83.5
Cbei_1839	altronate dehydratase	ND*	+118

Cbei_2200	mannonate dehydratase	ND*	+6.99
Cbei_2201	2-dehydro-3-deoxyphosphogluconate aldolase	ND*	+8.6
Cbei_2384	xylulokinase	ND*	+26.1
Cbei_2458	5-keto-4-deoxyuronate isomerase	ND*	+6.35
Cbei_4082	glucuronate isomerase	ND*	+5.3
Cbei_4083	mannitol dehydrogenase domain-containingprotein	ND*	+5.46
Cbei_4084	mannonate dehydratase	ND*	+2.81
Cbei_4457	L-arabinose isomerase	ND*	+14.1
Cbei_2383	xylose isomerase	ND*	+27.1
Pentose phosphate metabolism			
Cbei_0225	transketolase, central region	+3.19	+4.34
Cbei_0461	ribokinase-like domain-containing protein	ND*	+9.71
Cbei_0462	2-dehydro-3-deoxyphosphogluconate aldolase	ND*	+8.15
Cbei_1836	keto-hydroxyglutarate-aldolase/keto-deoxy-phosphogluconate aldolase	ND*	+92.5
Cbei_1837	ribokinase-like domain-containing protein	ND*	+105
Cbei_2076	ribose-phosphate pyrophosphokinase	ND*	+6.7
Cbei_2192	6-phosphogluconate dehydrogenase	ND*	+2.83
Cbei_2201	2-dehydro-3-deoxyphosphogluconate aldolase	ND*	+8.6
Cbei_2386	transaldolase	ND*	+6.87
Cbei_2387	transketolase	ND*	+11
Cbei_2432	phosphoribosylpyrophosphate synthetase	-2.76	+2.03
Cbei_3120	deoxyribose-phosphate aldolase	ND*	+2.22
Cbei_4453	transketolase	ND*	+10.8
Cbei_4454	transaldolase	ND*	+8.28
Cbei_4551	hypothetical protein	ND*	+5.72
Cbei_4644	transketolase	ND*	+11
Cbei_4645	transaldolase	ND*	+8.33

*: ND means NOT detected.

Table S5C. Fold change of amino acids biosynthesis / metabolism genes according to microarray analysis.

Cbei Symbol	Protein/Enzyme	Fold Change	
		Acidogenesis	Solventogenisis
Phenylalanine, tyrosine and tryptophan biosynthesis			
Cbei_4570	3-dehydroquinate dehydratase	+5.17	+2.97
Cbei_4575	3-phosphoshikimate 1-carboxyvinyltransferase	+2.2	+3.04
Cbei_1749	anthranilate synthase component I	+8.11	+7.03
Cbei_1750	glutamine amidotransferase of anthranilatesynthase	+8.75	+9.3
Cbei_1752	Indole-3-glycerol phosphate synthase	ND*	+9.36
Cbei_1753	phosphoribosylanthranilate isomerase	ND*	+15.1
Cbei_1755	tryptophan synthase subunit alpha	ND*	+7.22
Cbei_1754	tryptophan synthase subunit beta	ND*	+10.9
Cbei_4573	chorismate mutase	+2.67	+4.75
Cbei_4574	chorismate synthase	+2.38	+2.78
Cbei_4572	shikimate 5-dehydrogenase	+2.98	+2.86
Cbei_4571	shikimate kinase	+3.96	+2.82
Cbei_1295	hypothetical protein	ND*	+2.59
Alanine, aspartate and glutamate metabolism			
Cbei_5074	adenylosuccinate synthetase	-4.68	-2.91
Cbei_4516	argininosuccinate lyase	-24.2	-22.6
Cbei_4515	argininosuccinate synthase	-16.7	-26.3
Cbei_0028	carbamoyl phosphate synthase small subunit	-10.1	-5.88
Cbei_1034	asparagine synthase	-2.02	ND*
Cbei_1221	adenylosuccinate lyase	-3.25	-2.57
Cbei_1830	oxidoreductase	-2.62	ND*
Selenocompound metabolism			
Cbei_0055	methionyl-tRNA synthetase	ND*	+3.09
Cbei_1851	SufS subfamily cysteine desulfurase	ND*	+3.35

Cbei_3185	methionine--tRNA ligase	+5.39	+32.8
Cbei_4186	sulfate adenylyltransferase, large subunit	ND*	+3.61
Cbei_0577	cysteine synthase	ND*	+2.59
Cbei_0630	pyridoxal-5'-phosphate-dependent protein subunitbeta	ND*	+2.48
Cbei_4356	cysteine synthase A	-2.81	+2.38
Cbei_0428	S-adenosylmethionine synthetase	ND*	-3.52
Valine, leucine and isoleucine biosynethesis			
Cbei_0214	3-isopropylmalate dehydratase large subunit	+9.51	+4.84
Cbei_0216	3-isopropylmalate dehydrogenase	ND*	+14.4
Cbei_1043	branched-chain amino acid aminotransferase	ND*	+2.43
Cbei_0212	acetolactate synthase small subunit	+15.8	+3.24
Cbei_0213	alpha-isopropylmalate/homocitrate synthasefamily transferase	+19.9	+4.32
Cbei_0217	acetolactate synthase large subunit	ND*	+18.2
Cbei_4095	dihydroxy-acid dehydratase	ND*	+3.98
Cbei_4127	dihydroxy-acid dehydratase	ND*	+4.56
Cbei_0218	ketol-acid reductoisomerase	+3.83	+12.1
ABC transporter			
Cbei_0353	peptide ABC transporter periplasmic protein	ND*	+2.48
Cbei_0638	YaeC family lipoprotein	+2.09	+3.7
Cbei_1127	phosphate binding protein	-5.2	+4.38
Cbei_1128	phosphate ABC transporter permease	-2.92	+6.64
Cbei_1129	phosphate ABC transporter permease	-2.64	+8.82
Cbei_1130	phosphate ABC transporter ATPase	-2.34	+14.8
Cbei_1762	extracellular ligand-binding receptor	+5.57	+4.7
Cbei_1763	inner-membrane translocator	+6.33	+5.63
Cbei_1764	inner-membrane translocator	+4.5	+5.8
Cbei_1765	ABC transporter	+3.97	+5.87
Cbei_1766	ABC transporter	+5.86	+4.88
Cbei_1767	extracellular ligand-binding receptor	+3.62	+6.72

Cbei_1941	NMT1/THI5-like protein	+112	+30.9
Cbei_2357	extracellular solute-binding protein	ND*	+11.1
Cbei_2358	binding-protein-dependent transport system innermembrane protein	ND*	+17.2
Cbei_2377	periplasmic binding protein/LacI transcriptionalregulator	ND*	+6.4
Cbei_2382	monosaccharide-transporting ATPase	ND*	+2.79
Cbei_2394	ABC transporter	+2.53	+3.69
Cbei_2870	glycine/betaine ABC transportersubstrate-binding protein	+2.98	+12.2
Cbei_3096	ABC transporter	ND*	+14.3
Cbei_3539	glycine/betaine ABC transporter permease	ND*	+2.87
Cbei_3541	glycine/betaine ABC transportersubstrate-binding protein	ND*	+2.41
Cbei_3703	transport system permease	+4.93	+2.71
Cbei_3826	ABC transporter	ND*	+2.58
Cbei_3979	binding-protein-dependent transport system innermembrane protein	ND*	+2.14
Cbei_4190	sulfate ABC transporter ATPase	ND*	+4.71
Cbei_4191	sulfate ABC transporter permease	ND*	+5.75
Cbei_4192	sulfate ABC transporter permease	ND*	+4.38
Cbei_4194	sulfate ABC transporter substrate-bindingprotein	ND*	+8.71
Cbei_4448	monosaccharide-transporting ATPase	ND*	+73.3
Cbei_4449	ABC transporter	ND*	+38.9
Cbei_4450	monosaccharide-transporting ATPase	ND*	+10.8
Cbei_4928	extracellular solute-binding protein	+12.3	+18.1
Cbei_4929	peptide ABC transporter ATPase	+15.8	12.3
Cbei_4930	peptide ABC transporter ATPase	+12.1	+6.64
Cbei_4931	binding-protein-dependent transport system innermembrane protein	+11.8	+3.6
Cbei_4932	binding-protein-dependent transport system innermembrane protein	+9.66	+4.43
Cbei_4940	spermidine/putrescine ABC transporter ATPase	+147	+50.8
Cbei_5045	ABC transporter	ND*	+4.42
Cbei_5046	ABC transporter	ND*	+4.24
Glutathione metabolism			

Cbei_2192	6-phosphogluconate dehydrogenase	ND*	+2.83
Cbei_2737	leucyl aminopeptidase	ND*	+4.77
Cbei_4199	isocitrate dehydrogenase	+2.16	+3.45
Cbei_0777	glutathione peroxidase	ND*	+13.2
Cbei_0389	glutathione peroxidase	ND*	+6.93

*: ND means NOT detected.

Table S5D. Fold change of sigma factor & sporulation genes according to microarray analysis.

Cbei Symbol	Protein/Enzyme	Fold Change	
		Acidogenesis	Solventogenisis
Cbei_1302	ECF subfamily RNA polymerase sigma-24 factor	+6.96	+2.28
Cbei_0499	sporulation protein E	-2.92	-4.79
Cbei_1120	sigma-29; sporulation sigma factor SigE	-2.76	-4.24
Cbei_1122	sporulation protein YlmC/YmxH	-3.91	-3.21
Cbei_1210	sporulation protein YlmC/YmxH	+4.01	+1.28
Cbei_1218	stage V sporulation protein S	+2.53	+1.75
Cbei_1579	stage V sporulation protein D	ND*	-2.83
Cbei_5080	sporulation protease LonC	+2.55	-1.19
Cbei_1712	Spo0A protein	-1.50	-2.31
Cbei_0135	RNA polymerase factor sigma-70	-1.26	-2.09
Cbei_3569	RNA polymerase factor sigma-70	-14.28	-7.33
Cbei_3576	RNA polymerase factor sigma-70	-14.65	-7.60
Cbei_3675	RNA polymerase factor sigma-70	-2.50	-1.57
Cbei_4249	RNA polymerase sigma 28 subunit FliA/WhiG	+1.06	-4.04
Cbei_0595	RNA polymerase factor sigma-54	+1.40	+2.04
Cbei_0853	RNA polymerase sigma factor RpoD	-1.03	-3.39
Cbei_4947	sigma-K factor processing regulatory protein BofA	+3.76	+2.13
Cbei_0432	sigma-54 factor interaction domain-containing protein	-1.77	+3.76
Cbei_2039	sigma-54 dependent transcriptional regulator	-1.37	+3.40
Cbei_4686	sigma-54 factor interaction domain-containing protein	+5.44	+2.11
Cbei_4641	sigma-54 factor interaction domain-containing protein	+1.98	+5.30

*: ND means NOT detected.

Table S5E. Fold change of cell division proteins according to microarray analysis.

Cbei Symbol	Protein/Enzyme	Fold Change	
		Acidogenesis	Solventogenisis
Cbei_1117	cell division protein FtsA	ND*	-3.12
Cbei_1213	cell division protein FtsK	ND*	-3.88
Cbei_1173	signal recognition particle-docking protein FtsY	ND*	-3.01
Cbei_3037	ATP-dependent metalloprotease FtsH	+13.9	+16.6
Cbei_1575	cell division protein MraZ	ND*	-5.07
Cbei_4868	cell division ATP-binding protein FtsE	-2.77	-2.0
Cbei_0491	rod shape-determining protein MreB	ND*	-5.28
Cbei_0492	rod shape-determining protein MreC	ND*	-2.1
Cbei_0493	rod shape-determining protein MreD	ND*	-2.14
Cbei_0494	penicillin-binding protein, transpeptidase	ND*	-4.42
Cbei_0499	cell cycle protein	-2.92	-4.79
Cbei_3365	cell cycle protein RodA	-5.5	-3.66
Cbei_0425	rod shape-determining protein Mbl	-1.71	-1.71

*: ND means NOT detected.
